# Maize responsiveness to *Azospirillum brasilense*: Insights into genetic control, heterosis and genomic prediction

**DOI:** 10.1371/journal.pone.0217571

**Published:** 2019-06-07

**Authors:** Miriam Suzane Vidotti, Filipe Inácio Matias, Filipe Couto Alves, Paulino Pérez-Rodríguez, Gregório Alvarado Beltran, Juan Burgueño, José Crossa, Roberto Fritsche-Neto

**Affiliations:** 1 Genetics Department, “Luiz de Queiroz” College of Agriculture, University of São Paulo, Piracicaba, São Paulo, Brazil; 2 Department of Epidemiology & Biostatistics, Michigan State University, East Lansing, Michigan, United States of America; 3 Statistic Department, Colegio de Postgraduados, Texcoco, Estado de México, Mexico; 4 Biometrics and Statistics Unit, International Maize and Wheat Improvement Center (CIMMYT), Texcoco, Estado de Mexico, Mexico; Osmania University, INDIA

## Abstract

Several studies have shown differences in the abilities of maize genotypes to facilitate or impede *Azospirillum brasilense* colonization and to receive benefits from this association. Hence, our aim was to study the genetic control, heterosis effect and the prediction accuracy of the shoot and root traits of maize in response to *A*. *brasilense*. For that, we evaluated 118 hybrids under two contrasting scenarios: *i)* N stress (control) and *ii)* N stress plus *A*. *brasilense* inoculation. The diallel analyses were performed using mixed model equations, and the genomic prediction models accounted for the general and specific combining ability (GCA and SCA, respectively) and the presence or not of G×E effects. In addition, the genomic models were fitted considering parametric (G-BLUP) and semi-parametric (RKHS) kernels. The genotypes showed significant inoculation effect for five root traits, and the GCA and SCA were significant for both. The GCA in the inoculated treatment presented a greater magnitude than the control, whereas the opposite was observed for SCA. Heterosis was weakly influenced by the inoculation, and the heterozygosity and N status in the plant can have a role in the benefits that can be obtained from this Plant Growth-Promoting Bacteria (PGPB). Prediction accuracies for N stress plus *A*. *brasilense* ranged from 0.42 to 0.78, depending on the scenario and trait, and were higher, in most cases, than the non-inoculated treatment. Finally, our findings provide an understanding of the quantitative variation of maize responsiveness to *A*. *brasilense* and important insights to be applied in maize breeding aiming the development of superior hybrids for this association.

## Introduction

In recent years, several Plant Growth-Promoting Bacteria (PGPB) have been isolated, and their beneficial effects on the production of phytohormones and biological nitrogen fixation (N) have been identified [1,2]. The presence of these mechanisms permits certain strains to be used commercially as inoculants, which is a sustainable alternative to the use of chemical fertilizers and to mitigate biotic and abiotic stress. In this context, the species belonging to the genus is *Azospirillum* sp. have been extensively studied for agricultural purposes due to the excellent potential for response in association with cereal crops, such as maize [3]. The use of *Azospirillum* sp. in cropping areas increase up to 30% the grain yield and reduce up to 25% in N fertilizer needs [4,5]. Other beneficial effects of its use include the ability to modulate the root architecture, leading to a greater exploration of the soil and root expansion to deeper soil layers improving the access to water and nutrients [6,7].

The plant-PGPB association establishment involves complex mechanisms. In this sense, the genetic background of both host plant and bacteria plays a crucial role in the regulation of this partnership. Previous studies have shown differential responses among plant genotypes to *Azospirillum* sp. inoculation [8,9]. Genotypes may vary according to the amount and composition of the substances released in the exudates, as well the genes related to the plant defense mechanisms, directly affecting the responses to the inoculation [10,11].

Knowledge about the genetic control and inheritance of this association could help breeders to establish selection strategies for the partnership-related plant traits, which will enable the development of new cultivars with better responsiveness to inoculation. In this context, the diallel mating designs can be a useful approach for these studies, once it determines the genetic control and the relative importance of additive and non-additive genetic effects associated to agronomically important traits [12–14].

The evaluation of the maize responsiveness to PGPBs, as *Azospirillum* sp., is laborious and time-consuming. The use of genomic enabled approaches, as the genomic prediction [15], can be beneficial in this situations. Genomic prediction has been routinely implemented in maize breeding programs and is the object of study of several authors [16–19]. To our knowledge, there are no reports related to the genomic prediction of traits associated to the maize responsiveness to PGPB on the available literature.

Several studies have proposed the use of plant breeding to improve the interaction of plants with soil microorganisms [20–23]. So far, despite some progress being made in determining the effects of the *Azospirillum* sp. inoculation in maize [9,24], no studies about the maize genetic control for the responsiveness to the inoculation with this PGPB were reported, especially under N stress conditions, which is responsible for significant yield losses in many regions of the world [25]. In addition, although some authors speculate on the influence of maize heterosis on the efficiency of microorganism association [26,27], little is currently available about the effects of PGPB inoculation on this important phenomenon. Therefore, these knowledge could contribute to the selection of genotypes that are more efficient in the association with these microorganisms, thereby providing an effective technology for maize cultivation under low levels of N.

Hence, our objectives were *(i)* to identify the genetic control and inheritance of maize traits related to its responsiveness to *Azospirillum brasilense* under N stress, *(ii)* to verify the possible influence of heterosis and heterozygosity of maize on the benefits obtained from the association with this PGPB, and *(iii)* to compare the prediction accuracy of maize hybrids under inoculated and non-inoculated treatments through different prediction models.

## Materials and methods

### Plant material and *Azospirillum brasilense* inoculation

Nineteen inbred lines contrasting for Nitrogen Use Efficiency (NUE) were crossed in an incomplete diallel mating design (S1A Fig), without the reciprocals, to generate 118 single-cross maize hybrids. More information about the parental lines is presented in S2 Fig and Morosini et al. [28]. The commercial strain Ab-v5 of the Plant Growth-Promoting Bacteria (PGPB) *A*. *brasilense* was grown in dextrose yeast glucose sucrose (DYGS) liquid medium [29], while being shaken at 180 rpm, in the dark, at a temperature of 28ºC until reaching an optical density (OD) of 0.8. The bacterial cell concentration was adjusted to 10^−8^ colony-forming units (CFU) mL^-1^ by dilutions with the respective liquid medium. About 30 minutes before sowing, the inoculant was applied in plastic bags containing the non-sterile seeds of each genotype individually.

### Experimental design and phenotyping

Experiments were carried out in two years (2016 and 2017) under greenhouse conditions at Allogamous Plant Breeding Laboratory, “Luiz de Queiroz” College of Agriculture, University of São Paulo, Brazil (22°42'39"S; 47°38'09"W, altitude 540 m). The maize plants were grown in 3-L plastic pots containing unsterilized loam soil (S1 Table). To achieve optimal conditions for the bacterial biological N-fixation (low N condition), nitrogen fertilizer was not included [30]. Potassium chloride and super simple phosphate inputs according the crop needs were added to the soil. Three seeds were sown by pot with a posterior thinning to one seedling after germination. The experiments were conducted using the randomized complete block design with three replications by season. This was adopted due to the number of pots and a gradient of humidity and luminosity present in the greenhouse. In addition, the pots were allocated on two countertops. The two treatments consisted of N stress (control) and N stress plus *A*. *brasilense* inoculation. During the experiment, the average temperature was semi-controlled, with a maximum temperature of 33°C, and the water supply was provided individually per pot every other day or when necessary to maintain a well-watered condition. Supplementary luminosity was done with fluorescent lamps to establish a photoperiod of 12 hours of light. Parental inbred lines and hybrids were conducted under the same conditions but as individual experiments.

The phenotypes (in both years) were collected at the V7 stage of development (seven expanded leaves), about 35 days after emergence. Plant height (PH, cm) was measured, and the harvested shoot (leaves and stem) was dried in a forced draft oven at 60°C for 72 h to determinate the shoot dry mass (SDM, g). The roots were extracted and carefully rinsed with water to remove soil particles before being stored individually in plastic pots with a 25% ethanol solution for preservation. Images of each root were obtained using an Epson LA2400 scanner (2,400 dpi resolution) and after were analyzed by the WinRHIZO software (Reagent Instruments Inc., Quebec, Canada). This software provided measures of root average diameter (RAD, mm), root volume (RV, cm^3^), and length of a serial of root diameter classes. Lateral root length (roots formed from the axial roots—LRL, cm) and axial root length (comprising crow, seminal and primary roots—ARL, cm) were considered as roots fragments with a diameter class less than or equal to 0.5 mm and root fragments with a diameter class greater than 0.5 mm, respectively [31]. After the image analysis, the roots were dried to determine the root dry mass (RDM, g). Then, specific root length (SRL, cm g^-1^) and specific root surface area (SRSA, cm^2^ g^-1^) were calculated through the division of the total root length and the superficial area by RDM, respectively. All the measures were recorded individually by plant, thereby resulting in six replications per genotype for a total of 1,644 analyzed roots.

### Genotypic data

The genomic DNA of the parental inbred lines was extracted from leaf tissue at the V3 maize stage of development using a modified CTAB method [32] and was genotyped with the Affymetrix Axiom Maize Genotyping Array of 616,201 Single Nucleotide Polymorphism (SNP) markers [33]. We removed all markers with call rate lower than 95% and with heterozygous locus. The missing data imputation was performed by Beagle algorithm [34] available in the synbreed R package [35]. The matrix of hybrid genotypes was obtained by combining the parental lines genotypes. After, we discarded those markers with minor allele frequency (MAF) smaller than 0.05. Thus, a final SNP set of 65,225 and 52,215 for the inbred lines and hybrids, respectively.

### Diallel analysis

The diallel joint analysis across both treatments was performed for each trait by fitting the following model:

$$y=X_{E}\beta_{E}+X_{B}\beta_{B}+X_{C}\beta_{C}+X_{I}\beta_{I}+X_{EI}\beta_{EI}+Z_{G}u_{G}+Z_{H}u_{H}+Z_{GE}u_{GE}+Z_{HE}u_{HE}+Z_{GI}u_{GI}+Z_{HI}u_{HI}+\varepsilon$$

where ***y*** is the vector of hybrids phenotypes; ***β***_*E*_ is the vector of fixed effects of year; ***β***_*B*_ is the vector of the block within the year effect, considered as fixed; ***β***_*C*_ is the vector of the fixed effects of the countertop within block and year; ***β***_*I*_ is the vector of the fixed effects of inoculation; ***β***_*IE*_ is the vector of the fixed effect of the inoculation × year interaction; ***u***_*G*_ is the vector of random effects of general combining ability (GCA), with $u_{G}∼N(0,\sigma_{G}^{2}G)$, where $\sigma_{G}^{2}$ is the associated variance component and ***G*** is the associated additive relationship matrix from the parental inbred lines; ***u***_*H*_ is a vector of random effects of specific combining ability (SCA), with $u_{H}∼N(0,\sigma_{H}^{2}I_{H})$, where $\sigma_{H}^{2}$ is the associated variance component; ***u***_*GE*_ is the vector of random effects of GCA × year interaction, with $u_{GE}∼N(0,{\sigma_{GE}^{2}I}_{E}⊗G)$, where $\sigma_{GE}^{2}$ is the associated variance component; ***u***_*HE*_ is the vector of random effects of SCA × year interaction, with $u_{HE}∼N(0,\sigma_{HE}^{2}I_{E}⊗I_{H})$, where $\sigma_{HE}^{2}$ is the associated variance component; ***u***_*GI*_ is the vector of random effects of GCA × inoculation interaction, with $u_{GI}∼N(0,{\sigma_{GI}^{2}I}_{I}⊗G)$, where $\sigma_{GI}^{2}$ is the associated variance component; ***u***_***HI***_ is the vector of random effects of SCA × inoculation interaction, with $u_{HI}∼N(0,\sigma_{HI}^{2}I_{I}⊗I_{H})$, where $\sigma_{HI}^{2}$ is the associated variance component; ***ε*** is the vector of random residual effects, with $\varepsilon ∼N(0,\sigma_{\varepsilon }^{2}I)$. ***X***_***E***_, ***X***_***B***_, ***X***_***C***_, ***X***_***I***_, ***X***_***EI***_, ***Z***_***G***_, ***Z***_***H***_, ***Z***_***GE***_, ***Z***_***HE***_, ***Z***_***GI***_, and ***Z***_***HI***_ are the respective incidence matrices and ***I***_*H*_,***I***_*E*_,***I***_*H*_ are identity matrices of appropriate dimensions. ⊗ denotes the Kronecker product of matrices.

Individual diallel analyses for N stress and N stress plus *A*. *brasilense* inoculation were conducted employing the previous model disregarding the inoculation effect and its interactions effects with GCA and SCA. All analyses were carried out by the ASReml R package [36]. The synbreed R package [34] was used to obtain the ***G*** matrix by VanRaden method from the SNP set of inbred lines [37]. This dense matrix was posteriorly formatted as G-inverse, as required by ASReml, through a function included in MASS R package [38]. The Wald test implemented in ASReml was used to test the significance of the fixed effects. In turn, the significance of random effects was determined by the likelihood ratio test (LRT) using the asremlPlus R package [39]. The random effects from the diallel models were predicted as the Best Linear Unbiased Predictors (BLUPs), and their associated variance components were obtained using the Maximum Restricted Likelihood (REML) method. Both were automatically estimated by ASreml R package previously cited. Broad-sense heritability (*H*^2^) and narrow-sense heritability (*h*^2^) were estimated as:

$$H^{2}=(\sigma_{a}^{2}+\sigma_{d}^{2})/(\sigma_{a}^{2}+\sigma_{d}^{2}+\sigma_{\epsilon }^{2}),$$

$$h^{2}=\sigma_{a}^{2}∕(\sigma_{a}^{2}+\sigma_{d}^{2}+\sigma_{\epsilon }^{2})$$

where $\sigma_{a}^{2}$ is the additive genetic variance, $\sigma_{d}^{2}$ is the dominance genetic variance, and $\sigma_{\epsilon }^{2}$ is the residual variance. Genetic components were obtained as $\sigma_{a}^{2}=4\sigma_{G}^{2}$ and $\sigma_{d}^{2}=4\sigma_{H}^{2}$, where $\sigma_{G}^{2}$ and $\sigma_{H}^{2}$ are the GCA and SCA variances, respectively [40]. Considering that the genetic variance between single-cross progeny is $2\sigma_{a}^{2}+\sigma_{d}^{2}$, the relative importance of GCA and SCA was accessed by the Baker’s ratio as follows [41]:

$${BR=2\sigma }_{G}^{2}∕(2\sigma_{G}^{2}+\sigma_{H}^{2})$$

Principal Components (PCs) from the SCA values were obtained by treatment using the pre-installed R function prcomp(). The contribution of a variable *x* in accounting for the variability retained by PC1 and PC2 was expressed in percentage, according the following expression:

$${Cont}_{x}=[(C1*Eig1)+(C2*Eig2)]∕(Eig1+Eig2)$$

where *C*1 and *C*2 are the contribution of the variable *x* on PC1 and PC2 respectively; *Eig*1 and *Eig*2 are the amount of variation retained by PC1 and PC2, respectively. In addition, we dissected the relation between all measured traits, estimating the correlations among them. The igraph R package [42] was used to produce the network visualization plot from these results.

### Heterosis and heterozygosity estimates

Adjusted means of the hybrids and inbred lines in each treatment were obtained using the following model:

$$y=X_{E}\beta_{E}+X_{B}\beta_{B}+X_{C}\beta_{C}+X_{I}\beta_{I}+X_{EI}\beta_{EI}+X_{G}\beta_{G}+X_{GE}\beta_{GE}+\varepsilon$$

where ***y*** is the phenotypes of hybrids or inbred lines; ***β***_*E*_ is the vector of the fixed effects of year; ***β***_*B*_ is the vector of the block within year effect, considered as fixed; ***β***_*C*_ is the vector of the fixed effects of countertop within block and year; ***β***_*I*_ is the vector of fixed effects of inoculation; ***β***_*IE*_ is the vector of fixed effects of inoculation × year interactions; ***β***_*G*_ is the vector of fixed effects of the genotype; ***β***_*GE*_ is the fixed effects of genotype × year interaction; ***ε*** is the vector of random residual effects, with $\varepsilon ∼N(0,\sigma_{\varepsilon }^{2}I)$. ***X***_***E***_, ***X***_***B***_, ***X***_***C***_, ***X***_***I***_, ***X***_***EI***_, ***X***_***G***_, and ***X***_***GE***_ being the respective incidence matrices.

Mid-parent heterosis (MPH) and high-parent heterosis (HPH) were calculated for each hybrid for those traits with significant inoculation effect in the diallel joint analysis as:

MPH(%)=[(F1−MP)/MP]×100

HPH(%)=[(F1−BP)/BP]×100

where *F*1 is the mean performance of the hybrid; *MP* is the mid-parent value, given by (*P*1+*P*2)/2, where *P*1 and *P*2 are the mean performance of parental inbred line 1 and parental inbred line 2; *BP* is the mean performance of the better parental inbred line.

Furthermore, the individual heterozygosity level was estimated as the ratio between the number of heterozygous loci and the number of total markers from the genomic matrix. After, these values were correlated with the performance of the hybrids in the N stress and N stress plus *A*. *brasilensis*. Also, the heterozygosity was correlated with the difference of the hybrid performance in the two treatments (Δ), being Δ = *T*_2_−*T*_1_, where *T*_1_ and *T*_2_ are the hybrids adjusted means in N stress and N stress plus *A*. *brasilense*, respectively. Considering that the Δ in the biological sense is the change in the trait due to inoculation, this parameter for each hybrid was also correlated with the adjusted means in the N stress treatment.

### Genomic prediction

Parametric (G-BLUP) and semi-parametric (RKHS) prediction methods accounting for the general and specific combining abilities (GCA and SCA) were used to predict the performance of the single-crosses in the N stress and N stress plus *A*. *brasilense* inoculation scenarios. The fitted prediction models accounted for the genotype by the environment interaction effects (multi-environment, only variance G×E deviation model) or not (across environments). For the genomic prediction of the hybrids performance, we used a two-stage approach [43], where, in the first stage, the phenotypes were corrected for the experimental design effects, and in the second stage, the prediction models were fitted using the adjusted phenotypes. Genomic prediction methods were applied only for the traits with significant inoculation effect in the diallel joint analysis.

#### Across-environment GCA and SCA effects model

The model assumes the fixed effect of the environment and the random effects of the GCA of the parental inbred line and the SCA of the hybrid [44]. Here, each year was considered as an environment, being:

$$y=Z_{E}\beta_{E}+Z_{G}g+Z_{H}h+\varepsilon ,$$

where ***y*** is the vector of adjusted phenotypes; ***β***_*E*_ is the vector of environmental fixed effects; ***g*** is the vector of random effects of GCA with $g∼N(0,\sigma_{G}^{2}G)$, where $\sigma_{G}^{2}$ and ***G*** are the variance component and a variance covariance matrix associated with the GCA effects, respectively; ***h*** is the vector of random effects of SCA with $h∼N(0,\sigma_{H}^{2}H)$, where $\sigma_{H}^{2}$ and ***H*** are the variance component and the relationship matrix associated with the SCA effects, respectively; ***Z***_***E***_,***Z***_***G***_, and ***Z***_***H***_ are the respective incidence matrices; and ***ε*** is the vector of the residuals with $\varepsilon ∼N(0,\sigma_{\varepsilon }^{2}I)$.

This model was initially proposed to account for the GCA effects from inbred lines of two distinct heterotic groups [44]. However, in this study, we have the same inbred lines set composing the parental set one and two for the diallel mating design, thus, only one GCA effect must be modeled. For that, the incidence matrices for parental set one (***Z***_***p*1**_) and two (***Z***_***p*2**_) were computed separately, and ***Z***_***G***_ was obtained as ***Z***_***G***_ = ***Z***_***p*1**_+***Z***_***p*2**_.

We considered two covariance matrices: 1) ***GB*** = ***WW***′/*m*, where ***W*** is the centered and standardized matrix of the molecular markers for the inbreed lines, and *m* is the number of markers [45]; hereafter, we refer to the model that uses this matrix as GB; 2) The covariance matrix based on a Gaussian kernel obtained as $GK=exp(-hd_{ij}^{2})$, where $d_{ij}^{2}$ is the marker-based squared Euclidean distance between the individuals *i* and *j*; *h* is the bandwidth parameter. This parameter controls the decay rate of the ***GK*** values when the distance increases and in this study we considered *h* = 1. The use of semi-parametric kernels can be advantageous in genomic prediction models once potentially takes into account complex gene interactions (e.g., epistasis),which may increase the prediction accuracy[16].

For both kernels, the relationship matrix ***H*** was computed following the equation ***H***_(*ij*)(*i*′*j*′)_ = *C*_*ii*′_×*C*_*jj*′_, where ***H***_(*ij*)(*i*′*j*′)_ is the genetic covariance between two distinct hybrids; *C*_*ii*′_ and *C*_*jj*′_ are the entries from the parental covariance matrix (***GB*** or ***GK***) for group 1 and 2, respectively [44,46]. × is the Hadamard product.

#### Multi-environment, single variance G×E deviation model

We fitted the extended previous model by adding GCA and SCA × environment interactions, as proposed by Acosta-Pech et al.[46]:

$$y=Z_{E}\beta_{E}+Z_{G}g+Z_{H}h+u_{G}+u_{H}+\varepsilon$$

where ***u***_*G*_ is the vector of random interaction effects of GCA with the environment with $u_{G}∼N(0,\sigma_{GE}^{2}V_{G})$, where $\sigma_{GE}^{2}$ and ***V***_*G*_ are the relative variance component and the associated variance-covariance matrix; ***u***_*H*_ is the vector of random interaction effects of SCA with the environment with $u_{H}∼N(0,\sigma_{HE}^{2}V_{H})$, where $\sigma_{HE}^{2}$ and ***V***_*H*_ are the relative variance component and the associated variance-covariance matrix.

The ***V***_*G*_ and ***V***_*H*_ matrices were derived as $V_{G}={[Z}_{G}GZ_{G}^{\prime }]\times {[Z}_{E}Z_{E}\prime ]$ and $V_{H}={[Z}_{H}HZ_{H}^{\prime }]\times {[Z}_{E}Z_{E}\prime ].$ Our GBLUP model (*GB*+*G*×*E*) is equivalent to Acosta-Pech et al. [46]. Furthermore, we also tested the Gaussian kernel (*GK*+*G*×*E*).

#### Variance components and prediction accuracy

All the variance components were estimated by fitting the models using the Bayesian Generalized Linear Regression (BGLR) R package [47]. The results were based on 50,000 iterations after a burn-in period of 5,000 iterations. The mean posterior of variance components and standard deviation for GCA ($\sigma_{G}^{2}$), SCA ($\sigma_{H}^{2}$), GCA and SCA x environment interactions ($\sigma_{GE}^{2}$ and $\sigma_{GH}^{2}$), and residual variance ($\sigma_{\epsilon }^{2}$) were reported, with $\sigma_{GE}^{2}$ and $\sigma_{GH}^{2}$ being considered only in the (*GB*+*G*×*E*) and (*GK*+*G*×*E*) models.

The comparisons between the models were based on their prediction accuracies from cross-validation (CV) schemes simulating two prediction problems, as proposed by Burgueño et al. [48]. First, we assessed the prediction accuracy of the models considering that a set of hybrids was not evaluated in any of the environments (CV1). Second, we considered the problem of incomplete trials, where a set of hybrids are conducted only in part but not in all of the target environments (CV2). For both CV procedures, the hybrids were divided randomly into five groups, and four of them were used as the training set (TS) to estimate marker effects and to predict the phenotypes of individuals assigned to the fifth fold, referred to as the validation set (VS). The process was repeated 100 times for each model. For each TS-VS partition, the Pearson correlation was estimated, and the prediction accuracy was reported as the average of these values.

## Results

### Genetic correlations between traits

**Fig 1:**
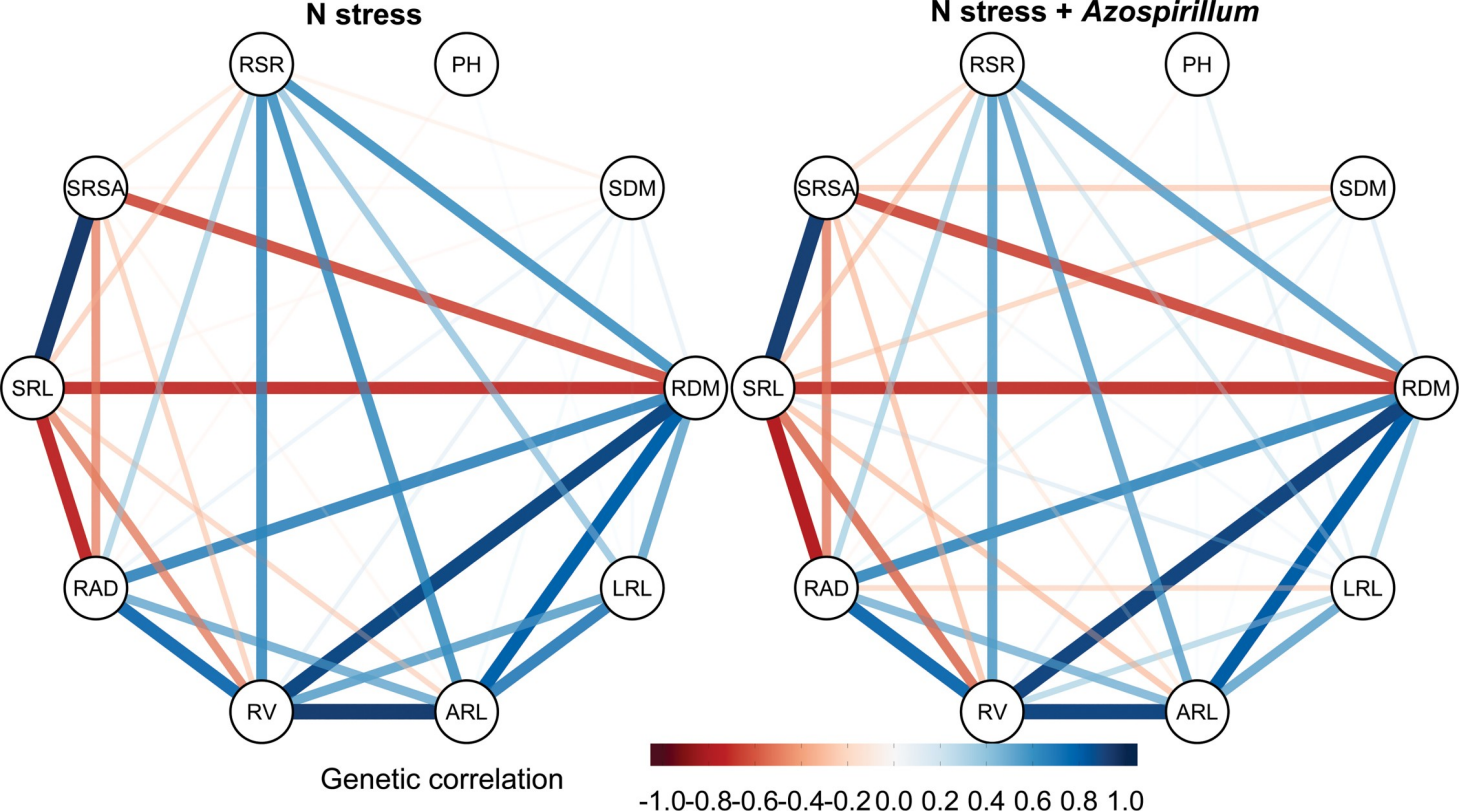
Network visualization of the genetic correlations among traits. The color variation of the lines indicate the direction (negative or positive) and the thickness indicate the magnitude. PH: plant height, SDM: shot dry mass, RDM: root dry mass, LRL: lateral root length, ARL: axial root length, RV: root volume, RAD: root average diameter, SRL: specific root length, SRSA: specific root surface area, and RSR: root shoot ratio.

The different degrees of the genetic correlations among the traits were revealed by the network (Fig 1). However, few substantial differences were observed among treatments (N stress and N stress plus *A*. *brasilense*), being, for most of the cases, the observed differences due changes to the correlation’s estimates magnitudes than to its direction. This is clear for the correlations between LRL and RV, ARL, RSR, and RDM, that were smaller in the inoculated treatment in comparison to the N stress condition. For both treatments, strong positive correlations were observed within the group of RDM, RV, RAD, and ARL. Additionally, the SRL and SRSA were positively correlated, however, these traits were negatively correlated with RDM, RV, and RAD.

### Importance of additive and non-additive gene action for the hybrid phenotype under the different treatments

In the diallel joint analysis performed across both treatments, the fixed effects of year and experimental design (block and countertop) were significant (*α*≤0.05) for most of the traits, indicating the importance of environmental control even in greenhouse conditions (Table 1). The *Y*×*I* effect (inoculation by year interaction) was not significant for any trait, suggesting that the responses of the genotypes to inoculation do not change differentially with the year. The *A*. *brasilense* inoculation effect was significant only for RDM, RV, RAD, SRL, and SRSA. Thus, all subsequent results and analyses were reported only for these five root traits.

**Table 1:** Joint diallel analyses of maize hybrids evaluated under N stress and N stress plus *Azospirillum brasilense* treatments.

Effects	PH	SDM	RDM	LRL	ARL	RV	RAD	SRL	SRSA	RSR
***Fixed***										
Year (Y)	1,484.0**	576.1**	29.3**	42.5**	1.6	11.6**	116.0**	11.7**	103.6**	818.5**
Block/Year	73.0**	4.0**	18.5**	27.8**	13.0*	63.4**	223.0**	88.0**	58.4**	22.8**
Countertop/Block	530.0**	164.8**	159.2**	185.6**	205.2**	182.6**	9.0	14.9*	20.5**	67.2**
Inoculation (I)	0	0.1	8.8**	0.2	1.2	10.3**	25.0**	11.7**	8.6**	3.4
Y x I	0	0.1	0.5	1.0	0	0	1.0	1.7	1.1	1.3
***Random***										
GCA	2.7	0.5	7.5**	12.4**	17.5**	16.4**	11.6**	7.9**	6.3*	6.1*
GCA x Y	-4.5 x 10^−7^	-3.5 x 10^−7^	0.9	7.4 x 10^−2^	1.8	1,6	2.1	4.3*	5.4*	19.8**
GCA x I	1.1 x 10^−2^	-2.3 x 10^−5^	5.0*	1.3 x 10^−5^	1.4	3.3	11.1**	6.9**	5.2*	1.7
SCA	0.3	2.1	1.0	-3.1 x 10^−6^	4.3*	3.1	3.2	6.3*	6.2*	2.8
SCA x Y	15.0**	7.8**	1.0	3.5	6.3 x 10^−2^	5.7 x 10^−3^	0.2	-1.1 x 10^−6^	8.4 x 10^−2^	0.2
SCA x I	-3.0 x 10^−5^	-1.5 x 10^−5^	-4.4 x 10^−5^	8.1 x 10^−6^	-3.2 x 10^−5^	-3.9 x 10^−5^	-4.3 x 10^−5^	-1.6 x 10^−5^	-4.0 x 10^−5^	-3.9 x 10^−5^

PH: plant height, SDM: shot dry mass, RDM: root dry mass, LRL: lateral root length, ARL: axial root length, RV: root volume, RAD: root average diameter, SRL: specific root length, SRSA: specific root surface area, and RSR: root shoot ratio. Significant at 5% (*) or 1% (**) level.

The most of the genetic variation observed among genotypes was due to the general combining ability (GCA) (Table 1). On the other hand, the specific combining ability (SCA) was significative only for SRL and SRSA. For these same two traits, significant effects of *GCA*×*Y* and *SCA*×*Y* were observed. Moreover, we verified significant *GCA*×*I* for all the traits, except for RV, whereas SCAxI was not detected for any trait.

**Fig 2:**
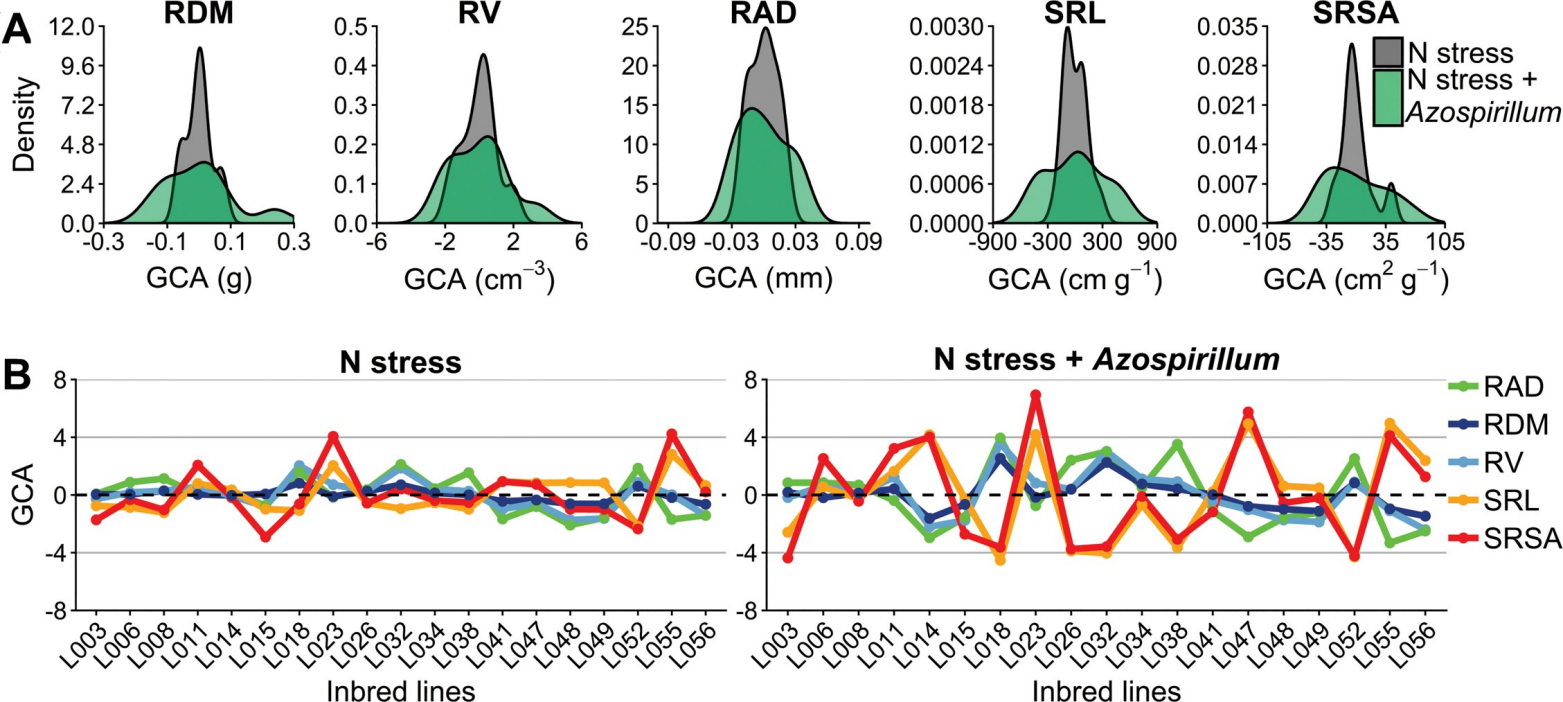
General combining ability (GCA) of the 19 parental inbred lines. (A) Density plots by trait. (B) Distribution by inbred lines (For this plot, the values of RDM, RAD, SRL and SRSA must be multiplied by 10^−1^, 10^−2^, 10^2^ and 10, respectively, to return to its correct magnitude). RDM: root dry mass, RV: root volume, RAD: root average diameter, SRL: specific root length, and SRSA: specific root surface area.

Concerning the variance components from the joint (Table 2) and individual (S1 and S2 Tables) diallel analyses, we found that the GCA variance (${\hat{\sigma }}_{G}^{2}$) contributed more significantly for the phenotypic variation in N stress plus *A*. *brasilense* than N stress (Fig 2, Table 2 and S4 Table).

**Table 2:** Estimatest of genetic parameters from the individual and joint diallel analysis.

Analysis	${\hat{\sigma }}_{G}^{2}$	${\hat{\sigma }}_{GY}^{2}$	${\hat{\sigma }}_{GI}^{2}$	${\hat{\sigma }}_{H}^{2}$	${\hat{\sigma }}_{HY}^{2}$	${\hat{\sigma }}_{HI}^{2}$	${\hat{\sigma }}_{\epsilon }^{2}$	${\hat{\sigma }}_{a}^{2}$	${\hat{\sigma }}_{d}^{2}$	${\hat{h}}^{2}$	${\hat{H}}^{2}$	BR
***RDM***												
N stress	6.24	1.62 x 10^−5^	-	3.06	2.31 x 10^−5^	-	159.74	24.95	12.22	0.13	0.19	0.80
N stress + *Azospirillum*	31.17	7.50	-	1.84	1.95 x 10^−5^	-	13.87	126.83	7.38	0.46	0.49	0.97
Joint	15.05	2.20	3.87	3.07	3.73	6.3 x 10^−5^	145.76	60.18	12.29	0.28	0.33	0.91
***RV***												
N stress	2.4	1.89 x 10^−6^	-	0.35	9.04 x 10^−7^	-	18.75	9.54	1.39	0.32	0.36	0.93
N stress + *Azospirillum*	6.40	0.70	-	0.63	1.05 x 10^−5^	-	16.99	25.62	2.55	0.56	0.62	0.95
Joint	3.92	0.27	0.40	0.63	3.12 x 10^−2^	1.12 x 10^−5^	17.75	15.66	2.53	0.44	0.51	0.92
***RAD***												
N stress	4.28	0.40	-	0.86	2.89 x 10^−6^	-	28.61	17.12	3.43	0.34	0.41	0.90
N stress + *Azospirillum*	11.86	0.39	-	0.35	1.33	-	30.74	47.44	1.40	0.60	0.61	0.99
Joint	6.51	0.71	1.49	1.16	0.30	1.37 x 10^−5^	29.78	26.04	4.62	0.43	0.50	0.92
***SRL***												
N stress	84.81	43.98	-	50.23	3.04 x 10^−4^	-	1,019.22	339.25	200.90	0.22	0.35	0.77
N stress + *Azospirillum*	260.43	0.40	-	2.50	9.33 x 10^−5^	-	1,077.83	1,041.70	10.00	0.51	0.51	1.00
Joint	134.40	32.84	32.49	50.39	4.60 x 10^−5^	1.01 x 10^−4^	993.97	537.60	201.55	0.31	0.42	0.84
***SRSA***												
N stress	1,529.70	1,085.88	-	1,024.40	7.00 x 10^−4^	-	17,058.29	6,118.79	4,097.59	0.22	0.37	0.74
N stress + *Azospirillum*	3,587.74	61.92	-	357.63	7.98 x 10^−4^	-	16,801.18	14,351.97	1,430.50	0.44	0.48	0.95
Joint	2,034.36	723.48	443.77	960.39	120.99	5.33 x 10^−3^	16,729.18	8,137.46	3,841.57	0.28	0.41	0.81

${\hat{\sigma }}_{G}^{2}$: GCA variance; ${\hat{\sigma }}_{GY}^{2}$: GCA x year variance; ${\hat{\sigma }}_{GI}^{2}$: GCA x inoculation variance; ${\hat{\sigma }}_{H}^{2}$: SCA variance; ${\hat{\sigma }}_{HY}^{2}$: SCA x year variance, ${\hat{\sigma }}_{HI}^{2}$: SCA x inoculation variance; ${\hat{\sigma }}_{\epsilon }^{2}$: residual error variance; ${\hat{\sigma }}_{a}^{2}$: additive genetic variance and ${\hat{\sigma }}_{d}^{2}$: dominance genetic variance, narrow-sense heritability (${\hat{h}}^{2}$), broad-sense heritability (${\hat{H}}^{2}$), and Baker’s ratio (BR) for root dry mass (RDM), root volume (RV), root average diameter (RAD), specific root length (SRL) and specific root surface area (SRSA). Variance components of RDM, RAD and SRL must be multiplied by 10^−3^, 10^−4^ and 10^3^, respectively, to return to its correct magnitude.

**Fig 3:**
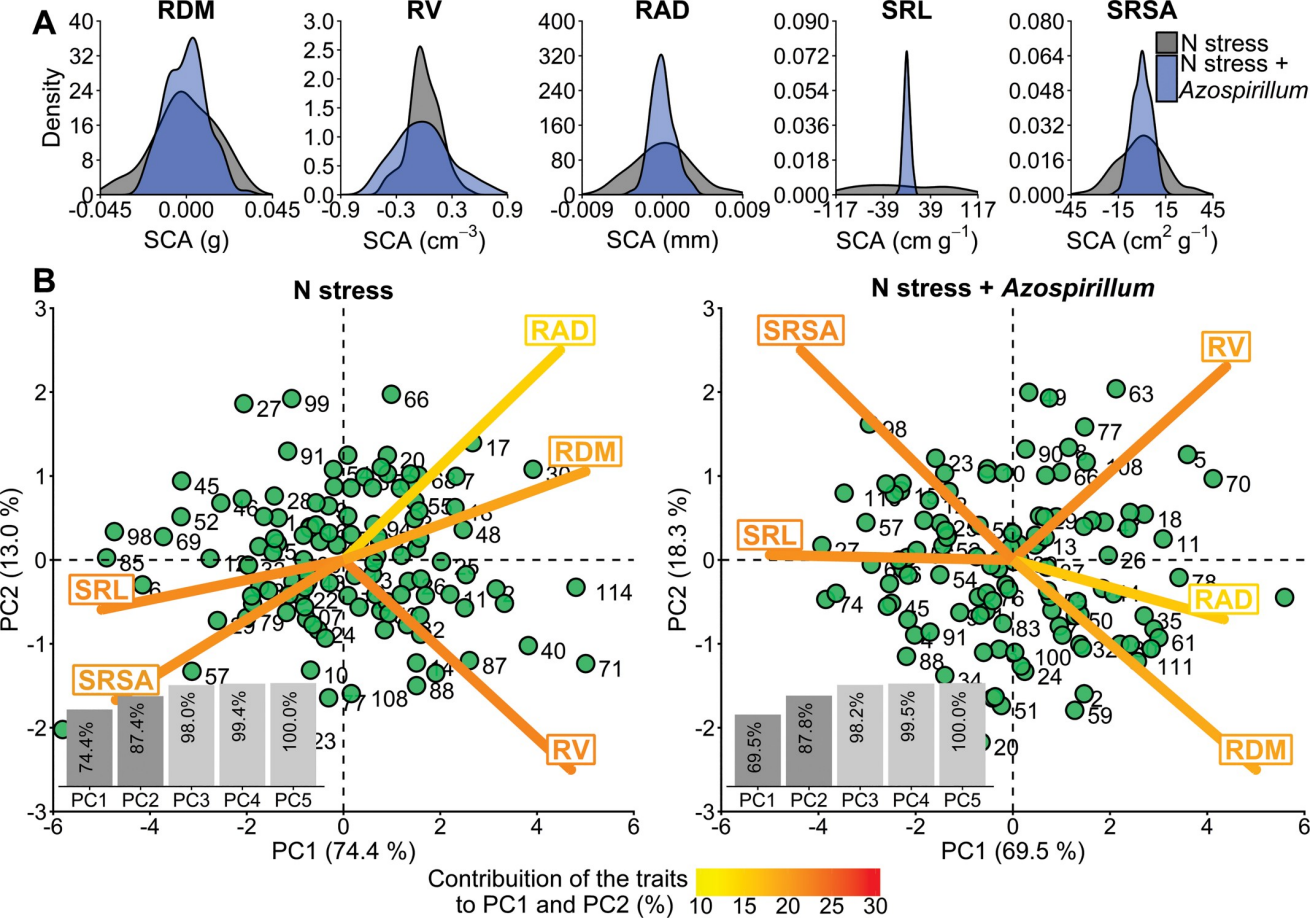
Specific combining ability (SCA) of the 118 maize hybrids. (A) Density plots by trait. (B) Principal Components (PCs), where each number corresponds to a different hybrid (more details are given in the S5 Table). RDM: root dry mass, RV: root volume, RAD: root average diameter, SRL: specific root length, and SRSA: specific root surface area.

For all traits, SCA variance estimates (${\hat{\sigma }}_{H}^{2}$) were higher under N stress than N stress plus *A*. *brasilense* (Table 2). For example, differences between non-inoculated and inoculated reached 95% for SRL, which was also evident on the variation of the SCA values among the hybrids (Fig 3A, S5 Table). The exception was only RV, where the values were 44.4% higher under the inoculated treatment. The Principal Components (PCs) of SCA values showed the genotypes and traits widely distributed throughout the projection space with substantial variation between the two treatments (Fig 3B). In general, each trait contributed approximately from 15% to 25% in the variation of PC1 and PC2. The first two principal components (PC1 and PC2) explained more than 87% of the observed variance in both treatments.

The Backer’s ratios were higher than 0.74 and 0.95 under N stress and N stress plus *A*. *brasilense*, respectively (Table 2), which suggest a considerable influence of GCA effects for the evaluated traits variation. The narrow-sense heritabilities estimates were higher for the inoculated treatment, ranging from 0.44 (SRSA) to 0.60 (RAD), whereas these estimates in the non-inoculated ranged from 0.13 (RDM) to 0.34 (RAD). Estimates of broad-sense heritability (${\hat{H}}^{2}$) were relatively close to narrow-sense heritability (${\hat{h}}^{2}$), where the smallest estimates were 0.19 (RDM) under N stress and 0.48 (SRSA) under N stress plus *A*. *brasilense*.

### Relation of heterosis and heterozygosity with root traits

**Fig 4:**
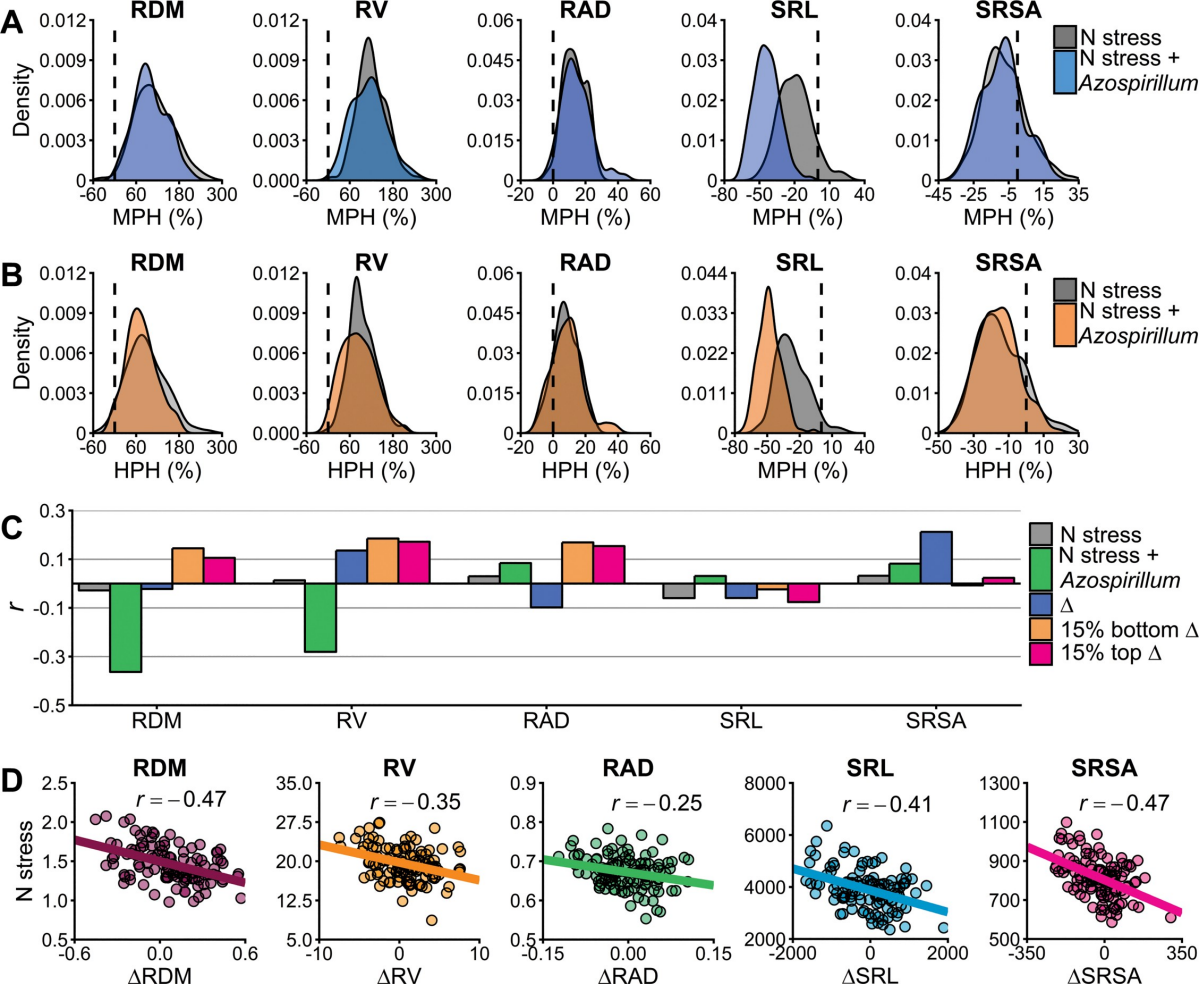
Heterosis effect and relationship with intrinsic root growth rates. (A) and (B) show the Mid-Parente Heterosis (MPH %) and the High-Parent Heterosis (HPH %), respectively. Black dashed line indicate the point where y is equal to zero. (C) Pearson correlation between hybrids adjusted means and genomic heterosigosity. Delta (Δ) is the change in root traits due to the inoculation (the difference between N stress plus *Azospirillum brasilense* inoculation and N stress) and thus, 15% bottom Δ and 15% top Δ represent the hybrids with smaller and higher responsiveness to inoculation, respectively. (D) Pearson correlation between hybrids adjusted means of N stress and delta. RDM: root dry mass, RV: root volume, RAD: root average diameter, SRL: specific root length, and SRSA: specific root surface area.

Heterosis was expressed relative to mid-parent (MPH) and high-parent (HPH) (Fig 4A and 4B). The distribution of the MPH estimates illustrates that only SRL exhibited pronounced differences between N stress and N stress plus *A*. *brasilense*, with average heterosis. For the other traits, no substantial variation was detected among the treatments. RDM and RV were the traits with greater heterosis over the mid-parent. Concerning HPH estimates, the root traits displayed a similar density pattern of MPH, except for RAD and SRSA.

Heterozygosity across the hybrids loci varied from 0.17 to 0.39, with a mean of 0.32. The correlation estimates between heterozygosity and adjusted means from the *A*. *brasilense* treatment was relatively low for all the traits (Fig 4C). Furthermore, the highest estimates were found for RDM (-0.36) and RV (-0.28). For the N stress treatment, all correlation values were less than 0.18. Changes due to inoculation (Δ) for each trait displayed low association with the hybrid’s genetic diversity and with the 15% bottom and top Δ hybrids, which represent the groups with smaller and larger responsiveness to inoculation to *A*. *brasilense*. Interestingly, the correlation values between the Δ and N stress ranged from -0.25 to -0.47, indicating that hybrids with greater root traits values in N stress tend to have less modulation of root architecture by *A*. *brasilense* inoculation (Fig 4D).

### Accuracy of predicting hybrid performance under inoculated and non-inoculated treatments

**Fig 5:**
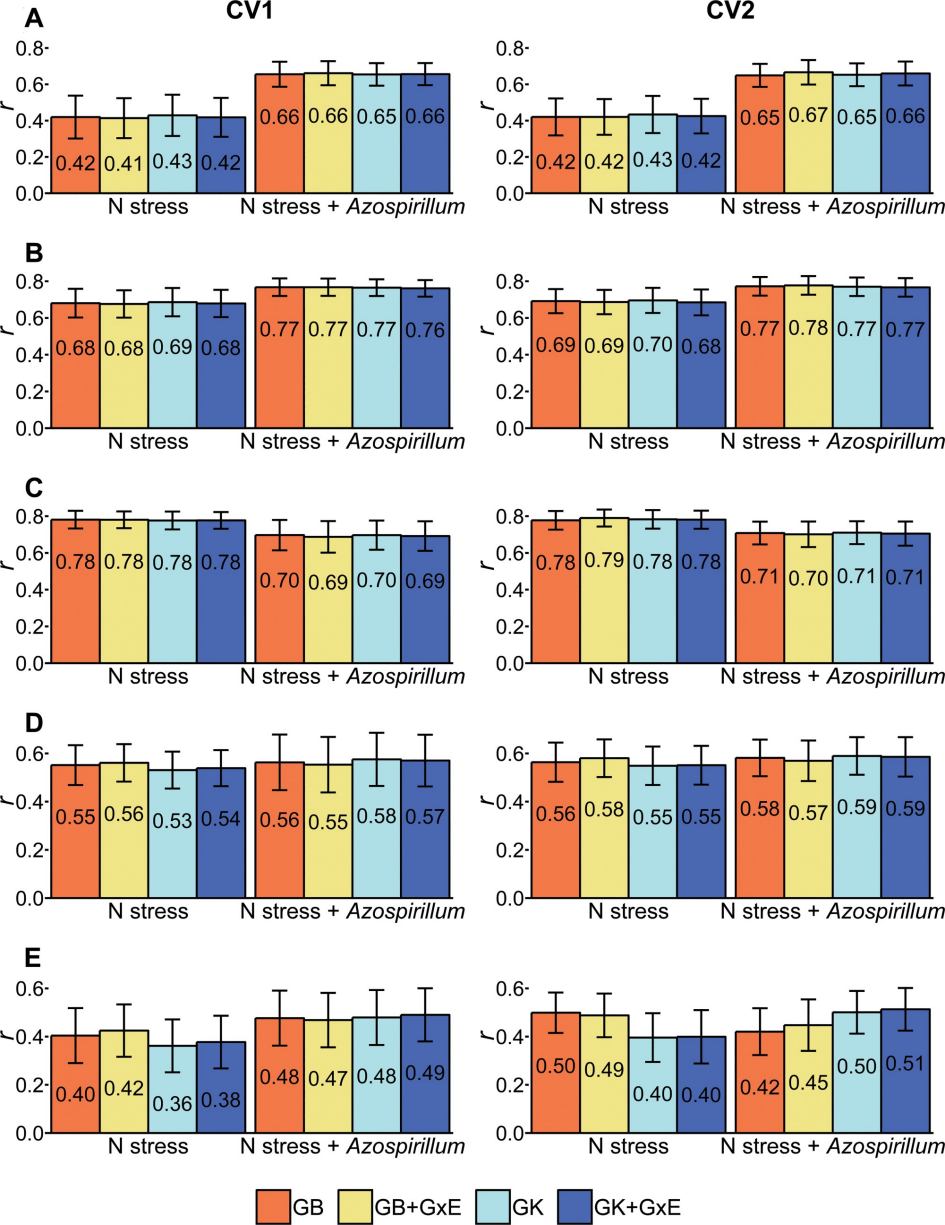
Prediction accuracies for across environment model with GBLUP (*GB*) and Gaussian Kernel (*GK*) and multi-environment model with GBLUP (*GB*+*G*×*E*) and Gaussian Kernel (*GK*+*G*×*E*) via cross-validation 1 and 2 methods. (A) root dry mass (RDM), (B) root volume (RV), (C) root average diameter (RAD), (D) specific root length (SRL), and (E) specific root surface area (SRSA).

Results of the prediction accuracy varied according to the root traits and treatments (**Fig 5**). For RDM and RV, the average prediction accuracy considering all the prediction models for N stress plus *A*. *brasilense* treatment were 0.66 and 0.77, respectively, and higher than N stress, for both CV methods. Additionally, a small increase in prediction accuracies were found for SRL on the inoculated treatment, with values ranging from 0.55 to 0.59. On the other hand, the prediction accuracies for RAD were reduced under N stress plus *A*. *brasilense*. For SRSA, the prediction accuracies varied according to the CV method. For example, in CV1 the prediction accuracies increased in the inoculated treatment, whereas in CV2 validation system, the results between the two treatments varied according to the prediction model.

Overall, high prediction accuracies were found under inoculated treatment, mainly for the traits related to the mitigation of N stress (RDM, RV, and RAD), ranging from 0.65 (RDM) to 0.78 (RV). Conversely, no substantial differences were observed between parametric and semi-parametric prediction models. Also, there was no advantage in modeling the *G*×*E* interaction. However, in general, the models with GBLUP displayed less residual variance that those with Gaussian kernel, thus indicating better adjustment of the models (S6, S7, S8, S9 and S10 Tables).

Concerning the cross-validations methods, for some traits (RDM, RV, RAD, and SRL) the prediction accuracies obtained in the CV2 validation approach slightly varied when compared to the CV1, which suggests that the recovered information among the environments was small. The highest gains in prediction accuracy when employing the CV2 over CV1 were found for the SRSA under N stress, especially in combination with GBLUP. However, surprisingly, we found a modest reduction of the prediction accuracy for the same trait when using the GBLUP model for prediction under N stress plus *A*. *brasilense*. In addition, for the same treatment, the opposite was found when the relationship between individuals was modeled using Gaussian kernel.

## Discussion

### Inheritable variance of root traits increases with *A*. *brasilense* inoculation

We performed individual and joint diallel analysis for 118 maize hybrids evaluated under N stress and N stress plus *A*. *brasilense* for a series of shoot and root related traits. From a total of ten traits, the RDM, RV, RAD, SRL, and SRSA were significantly affected by the bacterial inoculation. These results are consistent with those found under previous studies under similar growing conditions, indicating that the root growth promotion did not necessarily increase the shoot-related traits [7]. In turn, the variation observed for RDM, RV, and RAD reinforces *A*. *brasilense* capacity to modulate essential root traits by the production of phytohormones increasing the exploration of the soil and allow growth into deep soil layers, thus helping to mitigate stress conditions [3]. However, negative genetic correlations were observed among these traits and SRL and SRSA, which are related to phosphorus starvation tolerance [49]. Therefore, our results suggest that the selection of genotypes with enhanced responsiveness to *A*. *brasilense* inoculation should be specific for each stress condition.

A key finding of our study is the big importance of additive genetic effects for the phenotypic expression of root associated traits in N stress plus *A*. *brasilense* treatment in comparison to than N stress. Additive effects are inheritable and are a determinant factor for the evolutionary potential of species in natural conditions [50]. In this sense, the maize- *A*. *brasilense* interactions would enhance the plant’s ability to respond to environmental changes and persist over time. For example, in common bean, this PGPB can be vertically transmitted to successive plant generations demonstrating to be an effective inoculum in seed [51]. Conversely, in the context of plant breeding, the genetic gain with the selection under N stress plus *A*. *brasilense* inoculation could be more significant than N stress. However, we should be cautious with these results because in the diallel analysis is assumed absence of epistatic effects, and, it is known that epistasis can be manifested in several plant traits [52,53]. Moreover, according to Falconer and Mackay [54], additive genetic variance and additive-by-additive epistasis variance are responsible for the genetic value of each parental line (GCA) while other non-additive genetic variations are related to SCA. Thereby, a more substantial influence of GCA variance (and consequently the predominance of additive genetic variance) under inoculated treatment over non-inoculated possibly is due, in part, to the presence of an epistasis component. The importance of epistasis for underlying the complex genetic architecture of plant-pathogens interactions has been reported by several authors [55,56]. In this sense, further studies as genome-wide epistasis studies (GWES) could promote an understanding of the role and the relative importance of the genetic loci interactions for the differential ability of maize genotypes to establish an association with *A*. *brasilense*.

We also found low to moderate heritability estimates, which suggest a polygenic inheritance of the maize–*A*. *brasilense* association. This is consistent with the high number of genes that could be involved in the production of the root exudates, hormonal balance, and defense system that would modulate the plant bacterial colonization [9,24]. Additionally, although additive effects were greater than the dominance effects, both are involved in the genetic control of the maize root traits responsiveness to *A*. *brasilense* inoculation.

### Heterosis is variable across root traits but is weakly influenced by the bacterial inoculation

Heterosis, or hybrid vigor, is a phenomenon in which the hybrids often outperform their parents in yield, growth rate, biomass or stress tolerance [57]. Mid-parent heterosis (MPH) and high-parent heterosis (HPH) have been extensively exploited as a measure of heterosis [58,59]. We observed a very similar pattern of MPH and HPH distribution for the most of evaluated traits. Considering that high-parent and low-parent share close phenotypic values, similar estimates of mid-parent and high-parent are possible. This finding was expected due to the level of relatedness of our inbred lines set (S2 Fig). Therefore, our discussion focusses on merely using the term heterosis.

Although present in a range of traits, the heterotic responses can display variable levels among them [60,61]. The highest estimates were for RDM and RV, with the maximum values reaching 250%, suggesting a genetic divergence between the parental lines for these traits, which leads to the enhancement of the phenotypic expression. Furthermore, similarly to grain yield, RDM and RV can be the result of the multiplication of many others secondary traits, such the average root diameter, total root length, and surface area. Hence, the combination of quantitative variations can interact to produce higher heterosis, exceeding by more than double the mid- or high-parent heterosis, whereas the majority of the other traits display no more than 50% [62].

The analyses of heterosis showed positive, negative, or the combination of both directions. Considering that an increase of root biomass and expansion is often the primary driver for plant performance [63,64], the positive heterosis observed for RDM, RV, and RAD might confer better nutritional and water absorption when compared to the inbred lines. Conversely, the prevalence of negative heterosis for SRL and SRSA suggests a low genetic divergence in our inbred lines set for these traits. This is not surprising given that SRL and SRSA are more related to phosphorus stress tolerance, and our parental lines contrast in NUE.

The enhancement of growing conditions by the *A*. *brasilense* inoculation did not lead to pronounced variations in heterosis for most of the evaluated traits. However, heterotic responses in other maize traits, such as grain yield and leaf growth, can be correlated linearly with environmental quality [65,66]. Under these considerations, some explanations about our results are possible. First, the internal changes in the plant caused by the PGPB might have a relatively small effect on the genetic factors that trigger heterosis in comparison to other possible external sources. Second, depending on the trait, the heterosis over time may be relatively stable or variable during the plant lifecycle [59]. Therefore, in stages of development other than V7 heterosis, the results could be different between the treatments.

Interesting, in both treatments (inoculated and non-inoculated) the heterosis were similar. Even though four of the five traits showed lower ${\hat{\sigma }}_{d}^{2}$ estimates in N stress plus *A*. *brasilense* than under the N stress, similar heterosis levels were observed under both treatments. Advances in genetic and genomic studies have revealed that, in addition to traditional dominance and overdominance hypotheses, multiple causal mechanisms contribute to heterosis, including epistasis, epigenetic modification and small RNA activity [57]. As already mentioned, additive-by-additive epistatic effects might be present in the GCA effects. Thus, even with lower ${\hat{\sigma }}_{d}^{2}$ estimates, heterosis levels remained consistent for inoculated treatment. Under these considerations, additive-by-additive epistatic effects can play a prominent role in maize heterotic responsiveness under *A*. *brasilense* inoculation than dominance effects, such as observed in traits of bread wheat [67].

### Individual heterozygosity and N status in the plant can regulate the maize- *A*. *brasilense* partnership

Relations between genomic heterozygosity and plant fitness have been explored in several species, and, when studied at the individual level, these relations serve as a measure of individual genetic diversity [68,69]. Our analysis revealed modest negative correlations between heterozygosity and RDM and RV under N stress plus *A*. *brasilense* treatment. Most likely, the increase in the diversity of compounds released in the exudates because of high levels of heterozygosity results in an interaction with a more significant amount of soil microorganisms, leading to stimulation of other certain strains [26,70]. Hence, the competition between *A*. *brasilense* and a wide range of other microorganisms that have little or no effect on the plant could have resulted in a lower benefit due to inoculation. In addition, regarding host-pathogen systems, the lower individual genetic diversity can increase the susceptibility to infection [68,71]. Similarly, this could happen with *A*. *brasilense*, where certain heterozygous loci associated with plant immunity would actively control the extent of colonization by the bacteria and, consequently, the degree of beneficial results. Moreover, further studies are needed to better understand why some traits under inoculation treatment are more affected by heterozygosity level than other. We can speculate that the *A*. *brasilense* inoculation can stimulate certain heterozygous loci, which may affect differently the plant traits.

We also found negative correlations between performance under N stress and Δ (the difference between N stress plus *A*. *brasilense* and N stress treatments) for all evaluated traits. This correlation indicates that the average rate of increase due to inoculation tends to be higher in genotypes with worse performance under N stress effects. Thereby, traits related to NUE and the internal N status in the plant could be relevant for the development of more responsive maize hybrids to *A*. *brasilense* inoculation. Additionally, this observation reinforces the possible role of internal N metabolism regulating the association efficiency through the modulation of plant defense [11]. On the other hand, this may indicate that the plant breeding based on high N input can indirectly select less responsive plants to the PGPB association. Therefore, studies are needed to better understand the impact of plant breeding for N stress tolerance, for example, in the crosstalk and the efficiency association of *A*. *brasilense* and other PGPB.

### Hybrid performance under *A*. *brasilense* inoculation can be predicted with high accuracy

Our results showed high prediction accuracies for the majority of the traits evaluated under *A*. *brasilense* treatment in comparison to those traits observed under N stress. The high heritability estimates found for the inoculation condition could be a reason for these findings. Moreover, these results are consistent with those obtained in previous studies [16,72], in which the prediction accuracy under stress conditions tends to be lower than the non-limiting environments, especially for traits showing a very complex genetic architecture.

Although predictions with medium-to-high accuracy were found for both treatments in all tested genomic models, no substantial differences were observed between the use of parametric and semi-parametric methods (GB and GK, respectively). However, the limitations due to the small number of hybrids and the relatedness among our parental set may have influenced our results. For instance, in studies with larger panels of hybrids, superior overall performance of the nonlinear GK model relative to GBLUP has been observed [17,73]. In this sense, the use of GK proposed in our study, when applied to larger data sets, could show better results in comparison to the use of GB. The same explanation may be valid for the incorporation of *G*×*E* effects in the prediction models. The reduced number of environments in which our hybrids were tested and the greenhouse conditions (that reduce the action of environmental factors over the plant development) possibly resulted in high correlation among environments and led to a small occurrence of *G*×*E* interactions. In this sense, in our results, this modeling did not provide mainly an increase in the prediction accuracy for all traits and treatments. Our results reinforce the importance of prediction model-kernel combinations for both maize prediction under N stress plus *A*. *brasilense* and N stress, in addition to the possibility that they must be specific to each CV scheme for determined traits.

## Conclusions

We verified a quantitative inheritance of the maize responsiveness to *A*. *brasilense*, and that both additive and dominance genetic effects are involved the genetic control of this association. Furthermore, the heterozygosity and N status in the plant could influence the regulation of *A*. *brasilense* benefits to the plant. Prediction accuracies showed moderate to high values indicating an opportunity for genomic selection of traits related to the maize- *A*. *brasilense* partnership in the early stages of the plant development. Finally, our results may support possible plant breeding strategies to explore the genetic variability among maize genotypes relative to their differential ability to allow the colonization by *A*. *brasilense* and take advantage of this beneficial interaction.

## Supporting information

S1 Fig Information about the panel of 118 maize hybrids. (A) Scheme of obtaining from the crossing of 19 parental inbred lines in an incomplete diallel design (without the reciprocals), where the red squares indicate the hybrids evaluated and the gray squares represent the unrealized crosses. (B) Heatmap and dendogram of a kinship matrix estimated using Van Raden method based on 59,215 SNPs markers.
(TIF)

S2 Fig Panel structure analysis of the 19 maize parental inbred lines. Based on 65,225 SNPs markers, (A) the first two principal components, and (B) heatmap and dendogram of the kinship matrix estimated using the Van Raden method.
(TIF)

S3 Fig Boxplot of maize traits under N stress and N stress plus *Azospirillum brasilense*. PH: plant height, SDM: shot dry mass, RDM: root dry mass, LRL: lateral root length, ARL: axial root length, RV: root volume, RAD: root average diameter, SRL: specific root length, SRSA: specific root surface area, and RSR: root shoot ratio.
(TIF)

S1 Table Soil chemical and physic characteristics. (DOCX)

S2 Table Diallel analysis of maize hybrids evaluated under N stress. PH: plant height, SDM: shot dry mass, RDM: root dry mass, LRL: lateral root length, ARL: axial root length, RV: root volume, RAD: root average diameter, SRL: specific root length, SRSA: specific root surface area, and RSR: root shoot ratio. Significant at 5% (*) or 1% (**) level.
(DOCX)

S3 Table Diallel analysis of maize hybrids evaluated under N stress plus *Azospirillum brasilense*. PH: plant height, SDM: shot dry mass, RDM: root dry mass, LRL: lateral root length, ARL: axial root length, RV: root volume, RAD: root average diameter, SRL: specific root length, SRSA: specific root surface area, and RSR: root shoot ratio. Significant at 5% (*) or 1% (**) level.
(DOCX)

S4 Table Estimates of General Combining Ability (GCA) for 19 maize parental inbred lines. RDM: root dry mass, RV: root volume, RAD: root average diameter, SRL: specific root length, and SRSA: specific root surface area.
(DOCX)

S5 Table Estimates of Specific Combining Ability (SCA) for 118 maize hybrids. RDM: root dry mass, RV: root volume, RAD: root average diameter, SRL: specific root length, and SRSA: specific root surface area.
(DOCX)

S6 TableEstimates of variance components and standard deviation (in parentheses) from prediction models for root dry mass.$\sigma_{G}^{2}$: General Combining Ability (GCA); $\sigma_{H}^{2}$: Specific Combining Ability (SCA); $\sigma_{GE}^{2}$: GCA x environment interaction; $\sigma_{HE}^{2}$: SCA x environment interaction; $\sigma_{\epsilon }^{2}$: residual by fitting GBLUP (GB), *GBLUP*+*G*×*E* (*GB*+*G*×*E*), Gaussian Kernel (GK) and Gaussian Kernel + G×E (*GK*+*G*×*E*) models. The values must be multiplied by 10^−3^ to return to its correct magnitude.(DOCX)

S7 TableEstimates of variance components and standard deviation (in parentheses) from prediction models for root volume.$\sigma_{G}^{2}$: General Combining Ability (GCA); $\sigma_{H}^{2}$: Specific Combining Ability (SCA); $\sigma_{GE}^{2}$: GCA x environment interaction; $\sigma_{HE}^{2}$: SCA x environment interaction; $\sigma_{\epsilon }^{2}$: residual by fitting GBLUP (GB), *GBLUP*+*G*×*E* (*GB*+*G*×*E*)), Gaussian Kernel (GK) and Gaussian Kernel + G×E (*GK*+*G*×*E*) models.(DOCX)

S8 TableEstimates of variance components and standard deviation (in parentheses) from prediction models for root average diameter.$\sigma_{G}^{2}$: General Combining Ability (GCA); $\sigma_{H}^{2}$: Specific Combining Ability (SCA); $\sigma_{GE}^{2}$: GCA x environment interaction; $\sigma_{HE}^{2}$: SCA x environment interaction; $\sigma_{\epsilon }^{2}$: residual by fitting GBLUP (GB), *GBLUP*+*G*×*E* (*GB*+*G*×*E*), Gaussian Kernel (GK) and Gaussian Kernel + G×E (*GK*+*G*×*E*) models. The values must be multiplied by 10^−4^ to return to its correct magnitude.(DOCX)

S9 TableEstimates of variance components and standard deviation (in parentheses) from prediction models for specific root length.$\sigma_{G}^{2}$: General Combining Ability (GCA); $\sigma_{H}^{2}$: Specific Combining Ability (SCA); $\sigma_{GE}^{2}$: GCA x environment interaction; $\sigma_{HE}^{2}$: SCA x environment interaction; $\sigma_{\epsilon }^{2}$: residual by fitting GBLUP (GB), *GBLUP*+*G*×*E* (*GB*+*G*×*E*), Gaussian Kernel (GK) and Gaussian Kernel + G×E (*GK*+*G*×*E*) models.(DOCX)

S10 TableEstimates of variance components and standard deviation (in parentheses) from prediction models for specific root surface area.$\sigma_{G}^{2}$: General Combining Ability (GCA); $\sigma_{H}^{2}$: Specific Combining Ability (SCA); $\sigma_{GE}^{2}$: GCA x environment interaction; $\sigma_{HE}^{2}$: SCA x environment interaction; $\sigma_{\epsilon }^{2}$: residual by fitting GBLUP (GB), *GBLUP*+*G*×*E* (*GB*+*G*×*E*), Gaussian Kernel (GK) and Gaussian Kernel + G×E (*GK*+*G*×*E*) models.(DOCX)

S11 Table Adjusted means of root traits by maize genotype. (DOCX)
