# Supplementary material for: Maize responsiveness to Azospirillum brasilense: Insights into genetic control, heterosis and genomic prediction
Source: PLoS One. 2019 Jun 7;14(6):e0217571. doi: 10.1371/journal.pone.0217571 (PMC6555527; doi:10.1371/journal.pone.0217571)
Supplement: S1 Table — (DOCX) [file pone.0217571.s004.docx]

| **Parameters** | **Unity** | **Value** |
| --- | --- | --- |
| Soil pH (H_2_O) | - | 5.6 |
| Soil pH (CaCl_2_) | - | 4.7 |
| P (Mehlich) | mg.dm^-3^ | 72.5 |
| K | mg.dm^-3^ | 118.0 |
| Ca^+2^ | cmolc.dm·³ | 3.4 |
| Mg^+2^ | cmolc.dm·³ | 1.0 |
| Al^+3^ (KCl) | cmolc.dm·³ | 0.1 |
| Aluminium saturation (m) | % | 2.1 |
| Organic matter | g.dm·³ | 25.7 |
| Cation-exchange capacity (CEC) | cmolc.dm·³ | 8.6 |
| Clay | g kg·¹ | 226 |
| Silt | g kg·¹ | 167 |
| Sandy | g kg·¹ | 607 |
| Classification: *sandy loam soil* | | |

**S1 Table. Soil chemical and physic characteristics.**
