# Supplementary material for: Maize responsiveness to Azospirillum brasilense: Insights into genetic control, heterosis and genomic prediction
Source: PLoS One. 2019 Jun 7;14(6):e0217571. doi: 10.1371/journal.pone.0217571 (PMC6555527; doi:10.1371/journal.pone.0217571)
Supplement: S2 Table — PH: plant height, SDM: shot dry mass, RDM: root dry mass, LRL: lateral root length, ARL: axial root length, RV: root volume, RAD: root average diameter, SRL: specific root length, SRSA: specific root surface area, and RSR: root shoot ratio. Significant at 5% (*) or 1% (**) level. (DOCX) [file pone.0217571.s005.docx]

**S2 Table. Diallel analysis of maize hybrids evaluated under N stress.**

| **Effects** | **PH** | **SDM** | **RDM** | **LRL** | **ARL** | **RV** | **RAD** | **SRL** | **SRSA** | **RSR** |
| --- | --- | --- | --- | --- | --- | --- | --- | --- | --- | --- |
| ***Fixed*** |  |  |  |  |  |  |  |  |  |  |
| Year (Y) | 1,102.0** | 363.0** | 12.8** | 31.9** | 0.9 | 5.2* | 74.0** | 2.4 | 43.3** | 436.1** |
| Block/Year | 32.0** | 4.4 | 11.9* | 16.6** | 8.6 | 38.2** | 140.0** | 73.3** | 54.8** | 6.6 |
| Countertop/Block | 241.00** | 86.0** | 75.0** | 92.6** | 104.6** | 92.1** | 9.0* | 12.5 | 14.3* | 24.9** |
| ***Random*** |  |  |  |  |  |  |  |  |  |  |
| GCA | 4.3 x 10^-2^ | -4.0 x 10^-7^ | 3.3 | 7.2** | 13.5** | 11.0** | 8.7** | 3.1 | 2.8 | 0.5 |
| GCA x Y | -6.7 x 10^-7^ | 6.0 x 10^-2^ | -2.4 x 10^-6^ | -1.3 x 10^-5^ | -7.7 x 10^-6^ | -1.8 x 10^-6^ | 0.3 | 2.5 | 4.5* | 22.9** |
| SCA | 0.4 | 8.3 x 10^-7^ | 0.5 | -1.5 x 10^-5^ | 4.0 x 10^-2^ | 0.5 | 1.2 | 3.10.8 | 3.5 | 0.4 |
| SCA x Y | -1.9x10^-6^ | 0.8 | -7.8x10^-7^ | 2.2 x 10^-5^ | -1.3 x 10^-6^ | -2.3x10^-7^ | -7.0 x 10^-6^ | -1.6 x 10^-5^ | -1.1 x 10^-6^ | -9.7 x 10^-7^ |

PH: plant height, SDM: shot dry mass, RDM: root dry mass, LRL: lateral root length, ARL: axial root length, RV: root volume, RAD: root average diameter, SRL: specific root length, SRSA: specific root surface area, and RSR: root shoot ratio. Significant at 5% (*) or 1% (**) level.
