# Supplementary material for: Maize responsiveness to Azospirillum brasilense: Insights into genetic control, heterosis and genomic prediction
Source: PLoS One. 2019 Jun 7;14(6):e0217571. doi: 10.1371/journal.pone.0217571 (PMC6555527; doi:10.1371/journal.pone.0217571)
Supplement: S3 Table — PH: plant height, SDM: shot dry mass, RDM: root dry mass, LRL: lateral root length, ARL: axial root length, RV: root volume, RAD: root average diameter, SRL: specific root length, SRSA: specific root surface area, and RSR: root shoot ratio. Significant at 5% (*) or 1% (**) level. (DOCX) [file pone.0217571.s006.docx]

**S3 Table. Diallel analysis of maize hybrids evaluated under N stress plus *Azospirillum brasilense*.**

| **Effects** | **PH** | **SDM** | **RDM** | **LRL** | **ARL** | **RV** | **RAD** | **SRL** | **SRSA** | **RSR** |
| --- | --- | --- | --- | --- | --- | --- | --- | --- | --- | --- |
| ***Fixed*** |  |  |  |  |  |  |  |  |  |  |
| Year (Y) | 1068.0** | 376.9** | 20.5** | 18.4** | 0.5 | 6.7** | 47.0** | 11.9** | 69.4** | 432.3** |
| Block/Year | 30.0** | 1.3 | 9.8* | 19.1** | 5.9 | 27.4** | 109.0** | 36.0** | 22.1** | 24.0** |
| Countertop/Block | 297.0** | 90.9** | 89.3** | 100.4** | 107.7** | 95.9** | 3.0 | 7.8 | 13.5* | 55.9** |
| ***Random*** |  |  |  |  |  |  |  |  |  |  |
| GCA | 3.7* | 0.4 | 9.3** | 7.0** | 10.9** | 16.0** | 18.0** | 16.9** | 13.7** | 12.5** |
| GCA x Y | 0.4 | -3.6 x 10^-6^ | 2.8 | 3.9* | 9.5** | 2.6 | 0.2 | 3.2 x 10^-7^ | 2.4 x 10^-2^ | 2.3 |
| SCA | 0.3 | -1.5 x 10^-5^ | 0.2 | 3.1 x 10^-3^ | 3.2 | 1.5 | 7.7 x 10-^2^ | 9.0 x 10^-7^ | 0.5 | 3.5 x 10^-2^ |
| SCA x Y | -1.3 x 10^-6^ | -1.3 x 10^-5^ | -1.1 x 10^-6^ | 4.1 x 10^-4^ | -9.9 x 10^-7^ | -1.5 x 10^-5^ | 0.6 | -6.4 x 10^-7^ | -7.3 x 10^-7^ | 0.5 |

PH: plant height, SDM: shot dry mass, RDM: root dry mass, LRL: lateral root length, ARL: axial root length, RV: root volume, RAD: root average diameter, SRL: specific root length, SRSA: specific root surface area, and RSR: root shoot ratio. Significant at 5% (*) or 1% (**) level.
