# Supplementary material for: Maize responsiveness to Azospirillum brasilense: Insights into genetic control, heterosis and genomic prediction
Source: PLoS One. 2019 Jun 7;14(6):e0217571. doi: 10.1371/journal.pone.0217571 (PMC6555527; doi:10.1371/journal.pone.0217571)
Supplement: S4 Table — RDM: root dry mass, RV: root volume, RAD: root average diameter, SRL: specific root length, and SRSA: specific root surface area. (DOCX) [file pone.0217571.s007.docx]

**S4 Table. Estimates of General Combining Ability (GCA) for 19 maize parental inbred lines.**

| **Inbred line** | **N stress** | | | | |  | **N stress + *Azospirillum*** | | | | |
| --- | --- | --- | --- | --- | --- | --- | --- | --- | --- | --- | --- |
|  | **RDM** | **VR** | **RAD** | **SRL** | **SRSA** |  | **RDM** | **VR** | **RAD** | **SRL** | **SRSA** |
| L003 | 0.004 | -0.27 | 0.001 | -74.82 | -17.21 |  | 0.019 | -0.18 | 0.008 | -258.51 | -43.66 |
| L006 | 0.003 | 0.18 | 0.009 | -86.54 | -3.32 |  | -0.019 | 0.62 | 0.008 | 56.02 | 25.26 |
| L008 | 0.028 | 0.29 | 0.011 | -122.72 | -10.33 |  | 0.012 | 0.10 | 0.007 | -25.98 | -4.32 |
| L011 | 0.005 | 0.57 | 0.000 | 78.87 | 20.68 |  | 0.042 | 1.22 | -0.004 | 163.96 | 32.23 |
| L014 | -0.003 | -0.07 | 0.001 | 35.23 | -1.75 |  | -0.162 | -2.20 | -0.030 | 417.11 | 40.13 |
| L015 | 0.007 | -0.82 | -0.007 | -98.56 | -29.21 |  | -0.065 | -1.75 | -0.016 | -26.48 | -27.20 |
| L018 | 0.081 | 2.03 | 0.016 | -107.85 | -6.29 |  | 0.253 | 3.56 | 0.039 | -452.66 | -36.25 |
| L023 | -0.010 | 0.71 | -0.002 | 203.84 | 40.57 |  | -0.016 | 0.82 | -0.007 | 418.92 | 69.34 |
| L026 | 0.026 | 0.29 | 0.004 | -56.39 | -5.67 |  | 0.040 | 0.41 | 0.024 | -387.77 | -37.47 |
| L032 | 0.070 | 1.86 | 0.021 | -94.41 | 4.11 |  | 0.226 | 3.02 | 0.030 | -404.32 | -35.81 |
| L034 | 0.014 | 0.41 | 0.004 | -54.20 | -3.88 |  | 0.076 | 1.11 | 0.005 | -67.22 | -1.06 |
| L038 | 0.000 | 0.26 | 0.015 | -98.82 | -5.19 |  | 0.041 | 0.92 | 0.035 | -364.43 | -30.76 |
| L041 | -0.044 | -0.98 | -0.017 | 93.93 | 9.27 |  | 0.002 | -0.40 | -0.011 | 22.27 | -11.78 |
| L047 | -0.035 | -0.57 | -0.008 | 83.80 | 7.31 |  | -0.079 | -1.03 | -0.029 | 493.33 | 57.48 |
| L048 | -0.061 | -1.73 | -0.021 | 85.85 | -9.91 |  | -0.098 | -1.73 | -0.016 | 61.92 | -5.54 |
| L049 | -0.062 | -1.63 | -0.016 | 83.58 | -10.07 |  | -0.112 | -1.87 | -0.012 | 48.57 | -2.09 |
| L052 | 0.061 | 0.87 | 0.018 | -217.20 | -23.56 |  | 0.084 | 0.90 | 0.025 | -430.26 | -42.22 |
| L055 | -0.018 | 0.01 | -0.017 | 280.99 | 42.31 |  | -0.096 | -1.10 | -0.033 | 497.19 | 41.08 |
| L056 | -0.065 | -1.42 | -0.014 | 65.43 | 2.13 |  | -0.147 | -2.41 | -0.025 | 238.34 | 12.63 |

RDM: root dry mass, RV: root volume, RAD: root average diameter, SRL: specific root length, and SRSA: specific root surface area.
