# Supplementary material for: Maize responsiveness to Azospirillum brasilense: Insights into genetic control, heterosis and genomic prediction
Source: PLoS One. 2019 Jun 7;14(6):e0217571. doi: 10.1371/journal.pone.0217571 (PMC6555527; doi:10.1371/journal.pone.0217571)
Supplement: S5 Table — RDM: root dry mass, RV: root volume, RAD: root average diameter, SRL: specific root length, and SRSA: specific root surface area. (DOCX) [file pone.0217571.s008.docx]

**S5 Table. Estimates of Specific Combining Ability (SCA) for 118 maize hybrids.**

| **Nº** | **Cross** | **N stress** | | | | |  | **N stress + *Azospirillum*** | | | | |
| --- | --- | --- | --- | --- | --- | --- | --- | --- | --- | --- | --- | --- |
|  |  | **RDM** | **VR** | **RAD** | **SRL** | **SRSA** |  | **RDM** | **VR** | **RAD** | **SRL** | **SRSA** |
| 1 | L003xL006 | -0.013 | -0.15 | -0.0024 | 59.52 | 3.25 |  | -0.006 | -0.22 | -0.0001 | -0.25 | -0.61 |
| 2 | L003xL008 | 0.030 | 0.27 | 0.0039 | -149.88 | -20.47 |  | 0.015 | -0.03 | -0.0012 | -6.35 | -9.19 |
| 3 | L003xL014 | 0.011 | 0.07 | 0.0011 | -114.43 | -15.03 |  | -0.006 | -0.20 | -0.0005 | -2.24 | -3.04 |
| 4 | L003xL015 | 0.000 | -0.02 | -0.0011 | -1.69 | -1.60 |  | -0.017 | -0.43 | -0.0011 | 1.68 | 2.52 |
| 5 | L003xL018 | -0.001 | -0.05 | 0.0015 | -34.71 | -0.35 |  | 0.021 | 0.59 | 0.0031 | -6.54 | -3.64 |
| 6 | L003xL023 | -0.028 | -0.32 | -0.0050 | 266.52 | 32.57 |  | -0.003 | -0.22 | -0.0027 | 6.73 | 4.78 |
| 7 | L003xL026 | 0.012 | 0.06 | 0.0036 | -185.83 | -24.36 |  | 0.006 | -0.05 | 0.0001 | -3.66 | -5.05 |
| 8 | L003xL032 | 0.026 | 0.28 | 0.0067 | -167.35 | -15.59 |  | 0.005 | 0.39 | 0.0015 | -1.13 | 2.53 |
| 9 | L003xL034 | 0.004 | -0.07 | -0.0041 | -7.48 | -8.22 |  | -0.007 | -0.25 | -0.0008 | 1.73 | 0.10 |
| 10 | L003xL041 | -0.001 | 0.11 | -0.0006 | 102.98 | 16.35 |  | -0.005 | 0.21 | 0.0005 | 8.09 | 0.40 |
| 11 | L003xL047 | 0.014 | 0.19 | 0.0052 | -127.45 | -7.04 |  | 0.006 | 0.30 | 0.0036 | -9.60 | -5.25 |
| 12 | L003xL049 | -0.026 | -0.18 | -0.0039 | 132.59 | 20.49 |  | -0.006 | -0.07 | -0.0011 | 5.21 | 7.52 |
| 13 | L003xL052 | 0.008 | 0.01 | 0.0001 | -9.82 | 1.92 |  | 0.005 | 0.15 | -0.0001 | -0.49 | -0.86 |
| 14 | L006xL008 | 0.014 | 0.26 | 0.0032 | -33.40 | 1.76 |  | -0.001 | 0.15 | -0.0002 | 2.49 | 5.79 |
| 15 | L006xL011 | -0.014 | -0.14 | -0.0029 | 89.90 | 10.47 |  | -0.015 | -0.24 | -0.0001 | 6.37 | 9.68 |
| 16 | L006xL014 | -0.016 | -0.11 | 0.0015 | -7.56 | 6.67 |  | -0.008 | -0.11 | -0.0013 | 8.03 | 8.94 |
| 17 | L006xL015 | 0.019 | 0.03 | 0.0026 | -209.15 | -32.68 |  | 0.005 | -0.10 | 0.0005 | -4.03 | -5.65 |
| 18 | L006xL023 | 0.020 | 0.12 | 0.0013 | -151.28 | -25.06 |  | 0.015 | 0.51 | 0.0014 | -5.45 | -5.29 |
| 19 | L006xL026 | 0.001 | -0.05 | -0.0025 | 38.46 | -2.47 |  | 0.007 | 0.04 | -0.0004 | -2.80 | -4.42 |
| 20 | L006xL038 | 0.005 | -0.06 | -0.0007 | -108.49 | -20.04 |  | -0.009 | -0.54 | -0.0007 | -3.10 | -6.03 |
| 21 | L006xL047 | -0.008 | -0.09 | -0.0013 | 10.56 | 2.19 |  | 0.018 | 0.55 | 0.0005 | -3.91 | -5.03 |
| 22 | L006xL049 | -0.016 | -0.03 | 0.0021 | 41.97 | 16.07 |  | -0.001 | 0.02 | 0.0011 | -1.10 | -0.31 |
| 23 | L006xL052 | -0.005 | 0.16 | 0.0012 | 122.75 | 30.58 |  | -0.008 | 0.02 | -0.0004 | 5.51 | 8.97 |
| 24 | L008xL011 | 0.005 | 0.09 | -0.0033 | 70.71 | 7.76 |  | 0.006 | -0.11 | -0.0016 | -2.39 | -5.96 |
| 25 | L008xL015 | 0.019 | 0.15 | 0.0021 | -109.56 | -15.00 |  | 0.000 | 0.03 | -0.0016 | 11.88 | -0.72 |
| 26 | L008xL018 | 0.020 | 0.09 | 0.0012 | -69.32 | -5.38 |  | 0.002 | 0.15 | 0.0027 | -5.53 | -4.52 |
| 27 | L008xL023 | -0.032 | -0.39 | -0.0008 | 11.18 | 1.96 |  | -0.021 | -0.59 | -0.0015 | 9.53 | 11.62 |
| 28 | L008xL026 | -0.008 | -0.18 | -0.0045 | 52.87 | -0.22 |  | 0.004 | 0.16 | 0.0004 | -0.85 | -0.97 |
| 29 | L008xL032 | -0.017 | -0.15 | -0.0026 | 169.02 | 28.36 |  | 0.006 | 0.08 | 0.0008 | 0.03 | 1.03 |
| 30 | L008xL034 | 0.032 | 0.15 | 0.0048 | -250.99 | -39.26 |  | -0.005 | 0.12 | 0.0009 | 0.84 | 5.38 |
| 31 | L008xL041 | -0.017 | -0.11 | -0.0003 | 110.73 | 17.67 |  | -0.017 | -0.32 | -0.0005 | 3.67 | 6.69 |
| 32 | L008xL047 | 0.016 | 0.22 | 0.0003 | -35.98 | -5.22 |  | 0.005 | 0.04 | 0.0001 | -6.40 | -6.76 |
| 33 | L008xL048 | -0.012 | -0.10 | -0.0051 | 120.45 | 10.48 |  | 0.019 | 0.91 | 0.0015 | 0.84 | 6.13 |
| 34 | L008xL049 | -0.003 | -0.07 | -0.0026 | -5.10 | -0.04 |  | -0.011 | -0.44 | -0.0012 | -0.06 | -1.11 |
| 35 | L008xL052 | -0.021 | -0.13 | 0.0076 | -103.83 | -1.19 |  | 0.009 | 0.19 | 0.0019 | -8.07 | -9.46 |
| 36 | L008xL056 | -0.021 | -0.17 | -0.0015 | 74.36 | 6.41 |  | -0.018 | -0.49 | -0.0006 | 2.71 | 3.61 |
| 37 | L011xL014 | 0.009 | 0.03 | -0.0002 | -53.98 | -7.92 |  | 0.000 | 0.00 | 0.0011 | -2.65 | -1.44 |
| 38 | L011xL015 | 0.020 | 0.32 | 0.0027 | -52.17 | -1.14 |  | 0.002 | -0.09 | 0.0012 | -6.52 | -6.79 |
| 39 | L011xL018 | -0.002 | -0.05 | -0.0027 | 80.11 | 7.97 |  | 0.004 | 0.04 | -0.0004 | -0.01 | -0.26 |
| 40 | L011xL023 | 0.026 | 0.38 | 0.0086 | -175.46 | -13.68 |  | -0.008 | -0.18 | -0.0003 | 3.38 | 5.13 |
| 41 | L011xL026 | 0.006 | 0.06 | 0.0034 | -79.23 | -5.73 |  | 0.022 | 0.47 | 0.0001 | -3.51 | -5.39 |
| 42 | L011xL032 | 0.016 | 0.16 | -0.0001 | -4.46 | 0.13 |  | -0.002 | 0.04 | -0.0010 | 6.20 | 7.15 |
| 43 | L011xL034 | -0.010 | -0.04 | 0.0007 | 47.02 | 9.18 |  | 0.005 | 0.79 | 0.0018 | 2.04 | 9.32 |
| 44 | L011xL038 | 0.008 | 0.06 | 0.0003 | -11.51 | -4.52 |  | 0.016 | 0.23 | -0.0001 | -4.28 | -6.10 |
| 45 | L011xL047 | -0.038 | -0.40 | -0.0017 | 136.45 | 19.73 |  | -0.020 | -0.52 | -0.0005 | 4.77 | 4.54 |
| 46 | L011xL056 | -0.020 | -0.28 | -0.0043 | 130.19 | 9.47 |  | -0.006 | -0.15 | -0.0007 | 0.84 | -1.69 |
| 47 | L014xL015 | 0.014 | 0.15 | 0.0021 | -94.72 | -7.79 |  | 0.005 | 0.32 | 0.0018 | -4.61 | -2.95 |
| 48 | L014xL018 | 0.021 | 0.14 | 0.0029 | -174.22 | -18.86 |  | -0.001 | -0.03 | -0.0003 | 0.76 | 0.74 |
| 49 | L014xL023 | -0.004 | 0.00 | 0.0003 | 54.56 | 16.17 |  | 0.004 | 0.42 | 0.0010 | 3.07 | 6.57 |
| 50 | L014xL026 | 0.013 | 0.06 | -0.0016 | -50.97 | -6.73 |  | 0.002 | 0.02 | 0.0009 | -5.37 | -5.27 |
| 51 | L014xL032 | -0.005 | -0.01 | 0.0044 | -87.42 | -0.23 |  | -0.002 | -0.31 | -0.0012 | -2.86 | -5.81 |
| 52 | L014xL034 | -0.034 | -0.33 | -0.0030 | 141.72 | 23.45 |  | -0.008 | -0.17 | -0.0007 | 3.90 | 4.31 |
| 53 | L014xL038 | 0.000 | 0.14 | 0.0029 | -49.25 | 4.39 |  | 0.003 | 0.02 | 0.0002 | -4.00 | -2.60 |
| 54 | L014xL041 | -0.003 | -0.10 | -0.0030 | -35.09 | -12.39 |  | -0.001 | -0.19 | -0.0018 | 5.27 | 2.28 |
| 55 | L014xL047 | 0.008 | 0.05 | 0.0015 | -142.03 | -15.03 |  | 0.002 | 0.07 | -0.0011 | 4.89 | 1.47 |
| 56 | L014xL048 | -0.006 | -0.12 | -0.0059 | 137.01 | 8.95 |  | -0.009 | -0.19 | -0.0013 | 6.26 | 4.27 |
| 57 | L014xL049 | -0.011 | -0.11 | -0.0036 | 511.42 | 6.68 |  | -0.019 | -0.43 | -0.0005 | 7.98 | 9.85 |
| 58 | L014xL056 | -0.004 | 0.09 | 0.0007 | 57.41 | 12.41 |  | 0.009 | 0.20 | 0.0018 | -10.07 | -9.78 |
| 59 | L015xL018 | 0.009 | 0.08 | 0.0032 | -100.46 | -9.54 |  | 0.004 | -0.15 | -0.0001 | -6.97 | -9.67 |
| 60 | L015xL023 | -0.007 | -0.06 | -0.0011 | 56.60 | 4.66 |  | -0.016 | -0.45 | -0.0014 | 5.34 | 4.27 |
| 61 | L015xL032 | 0.012 | -0.07 | -0.0006 | -83.48 | -17.97 |  | 0.018 | 0.24 | 0.0008 | -9.07 | -10.80 |
| 62 | L015xL034 | -0.001 | -0.12 | -0.0051 | 78.77 | 4.01 |  | 0.002 | 0.07 | -0.0001 | -0.24 | 0.68 |
| 63 | L015xL038 | -0.004 | -0.02 | -0.0012 | 44.24 | 8.39 |  | 0.017 | 0.70 | 0.0014 | -1.60 | 2.64 |
| 64 | L015xL041 | -0.012 | -0.14 | 0.0018 | -76.95 | -9.23 |  | -0.008 | -0.18 | -0.0003 | 3.42 | 5.03 |
| 65 | L015xL047 | -0.013 | -0.10 | -0.0028 | 68.11 | 8.93 |  | -0.011 | -0.19 | -0.0010 | 7.91 | 8.97 |
| 66 | L015xL052 | 0.007 | -0.16 | -0.0011 | -145.53 | -26.78 |  | 0.010 | 0.39 | 0.0003 | 0.21 | 0.85 |
| 67 | L015xL055 | -0.034 | -0.24 | -0.0074 | 453.79 | 57.75 |  | -0.010 | -0.14 | -0.0003 | 2.73 | 5.72 |
| 68 | L015xL056 | -0.001 | -0.01 | 0.0045 | -156.95 | -15.67 |  | -0.008 | -0.35 | -0.0007 | -3.63 | -4.31 |
| 69 | L018xL023 | -0.018 | -0.30 | -0.0091 | 211.21 | 18.97 |  | -0.012 | -0.36 | -0.0022 | 7.56 | 7.06 |
| 70 | L018xL032 | -0.009 | 0.18 | 0.0041 | -34.74 | -1.60 |  | 0.021 | 0.65 | 0.0034 | -7.54 | -7.37 |
| 71 | L018xL038 | 0.052 | 0.49 | 0.0070 | -203.86 | -24.98 |  | -0.009 | -0.02 | -0.0002 | 3.46 | 7.10 |
| 72 | L018xL041 | 0.007 | 0.06 | -0.0011 | 13.13 | 2.34 |  | 0.010 | 0.11 | -0.0008 | -1.83 | -3.34 |
| 73 | L018xL055 | -0.008 | -0.06 | 0.0005 | 41.82 | 9.89 |  | 0.001 | 0.14 | 0.0003 | -1.92 | 0.61 |
| 74 | L018xL056 | 0.000 | -0.05 | -0.0026 | 101.48 | 9.68 |  | -0.014 | -0.49 | -0.0035 | 10.70 | 6.57 |
| 75 | L023xL026 | 0.026 | 0.21 | 0.0023 | -122.39 | -18.55 |  | -0.011 | -0.30 | 0.0008 | -0.09 | 0.81 |
| 76 | L023xL032 | 0.005 | 0.16 | 0.0025 | -61.93 | -3.50 |  | -0.004 | -0.12 | -0.0005 | 0.21 | -0.04 |
| 77 | L023xL034 | 0.002 | 0.15 | 0.0014 | 95.02 | 18.88 |  | 0.009 | 0.46 | 0.0018 | -0.36 | 2.03 |
| 78 | L023xL038 | 0.001 | 0.13 | 0.0044 | -30.13 | 2.76 |  | 0.011 | 0.40 | 0.0025 | -10.19 | -9.03 |
| 79 | L023xL041 | -0.010 | -0.04 | -0.0046 | 164.99 | 15.48 |  | 0.004 | 0.22 | 0.0007 | -1.95 | 0.12 |
| 80 | L023xL047 | -0.004 | -0.09 | 0.0005 | -63.12 | -6.97 |  | 0.013 | 0.18 | 0.0002 | -6.92 | -9.87 |
| 81 | L023xL048 | 0.002 | 0.10 | 0.0010 | -2.42 | 3.88 |  | 0.006 | 0.10 | 0.0003 | -5.00 | -5.88 |
| 82 | L023xL049 | 0.023 | 0.24 | 0.0043 | -102.42 | -12.49 |  | 0.006 | -0.05 | -0.0010 | 1.54 | -1.38 |
| 83 | L023xL055 | -0.005 | 0.03 | 0.0008 | 71.91 | 13.23 |  | -0.005 | -0.24 | 0.0002 | -2.44 | -1.03 |
| 84 | L023xL056 | -0.004 | -0.04 | -0.0061 | 156.55 | 10.96 |  | 0.011 | 0.48 | 0.0007 | 2.94 | 4.85 |
| 85 | L026xL032 | -0.037 | -0.44 | -0.0051 | 276.27 | 38.26 |  | -0.004 | 0.13 | 0.0016 | -1.35 | 0.15 |
| 86 | L026xL038 | -0.013 | 0.00 | 0.0015 | 52.56 | 14.87 |  | -0.011 | -0.12 | -0.0009 | 4.48 | 6.81 |
| 87 | L026xL047 | 0.026 | 0.33 | 0.0040 | -91.90 | -7.16 |  | -0.012 | -0.32 | -0.0012 | 5.07 | 4.70 |
| 88 | L032xL034 | 0.023 | 0.28 | 0.0017 | -6.85 | 1.98 |  | -0.009 | -0.49 | -0.0021 | 3.90 | 1.03 |
| 89 | L032xL038 | 0.019 | 0.06 | -0.0031 | -15.86 | -9.39 |  | -0.003 | -0.07 | 0.0000 | 0.21 | 0.50 |
| 90 | L032xL047 | 0.017 | 0.19 | 0.0023 | -60.52 | -4.51 |  | 0.007 | 0.34 | -0.0001 | 1.81 | 4.48 |
| 91 | L032xL052 | -0.004 | -0.24 | -0.0045 | 4.53 | -5.33 |  | -0.007 | -0.34 | -0.0018 | 3.04 | 0.76 |
| 92 | L034xL041 | 0.003 | 0.00 | 0.0027 | -124.11 | -16.57 |  | 0.014 | 0.16 | 0.0005 | -7.81 | -9.71 |
| 93 | L034xL047 | 0.010 | 0.18 | 0.0057 | -52.15 | 1.36 |  | 0.007 | 0.01 | 0.0009 | -4.01 | -4.81 |
| 94 | L034xL049 | 0.011 | 0.03 | -0.0024 | -61.56 | -11.21 |  | -0.013 | -0.35 | -0.0017 | 6.29 | 4.71 |
| 95 | L034xL052 | 0.010 | 0.11 | -0.0001 | -24.22 | -1.33 |  | 0.002 | 0.04 | 0.0027 | -6.76 | -6.18 |
| 96 | L034xL055 | 0.001 | 0.02 | -0.0012 | 34.19 | 3.07 |  | 0.006 | -0.12 | -0.0016 | -1.83 | -5.65 |
| 97 | L034xL056 | -0.025 | -0.15 | 0.0016 | 47.91 | 13.06 |  | 0.008 | 0.04 | -0.0010 | 1.15 | -0.39 |
| 98 | L038xL047 | -0.043 | -0.42 | -0.0048 | 254.59 | 32.53 |  | -0.010 | -0.16 | -0.0009 | 10.32 | 14.46 |
| 99 | L038xL049 | -0.025 | -0.32 | 0.0006 | -66.78 | -3.80 |  | 0.004 | 0.26 | 0.0018 | -4.26 | -1.80 |
| 100 | L038xL052 | 0.001 | -0.03 | -0.0022 | 64.69 | 7.75 |  | 0.001 | -0.20 | -0.0007 | -1.73 | -4.26 |
| 101 | L038xL055 | -0.011 | -0.04 | 0.0021 | -43.52 | -2.86 |  | -0.020 | -0.54 | -0.0017 | 13.47 | 3.08 |
| 102 | L038xL056 | 0.013 | 0.08 | 0.0003 | -114.70 | -18.78 |  | 0.011 | 0.16 | 0.0015 | -9.71 | -10.86 |
| 103 | L041xL047 | -0.004 | -0.06 | -0.0062 | 155.91 | 10.10 |  | 0.000 | -0.31 | -0.0013 | -2.04 | -0.60 |
| 104 | L041xL049 | -0.018 | -0.10 | 0.0016 | 13.85 | 7.38 |  | 0.002 | 0.23 | 0.0017 | -4.22 | -1.58 |
| 105 | L041xL056 | 0.005 | 0.01 | 0.0014 | -119.51 | -21.25 |  | -0.004 | -0.09 | 0.0002 | -0.99 | -0.90 |
| 106 | L047xL048 | -0.011 | -0.08 | -0.0004 | -46.53 | -6.22 |  | 0.000 | -0.08 | -0.0011 | 0.94 | -1.45 |
| 107 | L047xL052 | -0.009 | -0.03 | -0.0005 | 88.53 | 16.16 |  | -0.001 | 0.00 | 0.0003 | -0.19 | 1.20 |
| 108 | L047xL055 | 0.020 | 0.20 | -0.0021 | 127.78 | 4.91 |  | 0.017 | 0.53 | 0.0000 | -0.09 | -0.39 |
| 109 | L047xL056 | 0.003 | -0.03 | -0.0008 | -74.99 | -14.11 |  | -0.018 | -0.30 | 0.0003 | 8.37 | 9.82 |
| 110 | L048xL049 | -0.001 | -0.05 | 0.0005 | -118.39 | -14.36 |  | -0.010 | -0.40 | 0.0000 | -3.60 | -4.51 |
| 111 | L048xL052 | 0.022 | 0.11 | -0.0012 | -90.27 | -17.92 |  | 0.017 | 0.14 | 0.0003 | -7.73 | -11.23 |
| 112 | L048xL055 | -0.021 | -0.23 | -0.0032 | 98.14 | 5.72 |  | -0.011 | -0.22 | 0.0010 | 0.21 | 3.31 |
| 113 | L048xL056 | -0.018 | -0.06 | -0.0005 | 100.22 | 15.31 |  | -0.011 | -0.27 | -0.0023 | 12.19 | 10.50 |
| 114 | L049xL052 | 0.033 | 0.39 | 0.0091 | -275.25 | -26.60 |  | 0.001 | 0.16 | -0.0004 | 1.25 | 0.13 |
| 115 | L049xL056 | -0.008 | -0.02 | -0.0007 | -16.57 | -0.61 |  | 0.032 | 0.69 | 0.0024 | -14.58 | -15.72 |
| 116 | L052xL055 | 0.013 | 0.02 | -0.0015 | -92.51 | -21.00 |  | 0.001 | -0.07 | -0.0006 | 0.77 | 1.50 |
| 117 | L052xL056 | -0.007 | -0.11 | -0.0013 | -6.35 | -2.06 |  | -0.022 | -0.41 | 0.0001 | 3.77 | 6.97 |
| 118 | L055xL056 | 0.012 | 0.20 | 0.0024 | -66.53 | -10.20 |  | 0.008 | 0.33 | 0.0001 | 0.53 | 1.93 |

RDM: root dry mass, RV: root volume, RAD: root average diameter, SRL: specific root length, and SRSA: specific root surface area.
