# Supplementary material for: Maize responsiveness to Azospirillum brasilense: Insights into genetic control, heterosis and genomic prediction
Source: PLoS One. 2019 Jun 7;14(6):e0217571. doi: 10.1371/journal.pone.0217571 (PMC6555527; doi:10.1371/journal.pone.0217571)
Supplement: S6 Table — σG2: General Combining Ability (GCA); σH2: Specific Combining Ability (SCA); σGE2: GCA x environment interaction; σHE2: SCA x environment interaction; σϵ2: residual by fitting GBLUP (GB), GBLUP+G×E (GB+G×E), Gaussian Kernel (GK) and Gaussian Kernel + G×E (GK+G×E) models. The values must be multiplied by 10−3 to return to its correct magnitude. (DOCX) [file pone.0217571.s009.docx]

**S6 Table. Estimates of variance components and standard deviation (in parentheses) from prediction models for root dry mass**. The values must be multiplied by 10^-3^ to return to its correct magnitude.

| **Treatment** | $\sigma_{G}^{2}$ | $\sigma_{H}^{2}$ | $\sigma_{GE}^{2}$ | $\sigma_{HE}^{2}$ | $\sigma_{\epsilon}^{2}$ |
| --- | --- | --- | --- | --- | --- |
| ***GB*** |  |  |  |  |  |
| N stress | 6.03 (2.32) | 10.95 (3.59) | - | - | 5.25 (5.65) |
| N stress + *Azospirillum* | 18.12 (6.53) | 10.77 (3.16) | - | - | 47.76 (5.07) |
| ***GB + G***$\boldsymbol{\times}$***E*** |  |  |  |  |  |
| N stress | 4.33 (1.94) | 8.48 (3.26) | 3.78 (1.55) | 8.98 (3.92) | 45.58 (6.11) |
| N stress + *Azospirillum* | 13.36 (5.98) | 8.48 (2.96) | 6.97 (3.03) | 9.02 (3.23) | 37.91 (5.08) |
| ***GK*** |  |  |  |  |  |
| N stress | 0.856 (0.75) | 10.45 (4.53) | - | - | 54.96 (5.82) |
| N stress + *Azospirillum* | 15.77 (8.16) | 11.49 (6.34) | - | - | 49.30 (5.16) |
| ***GK + G***$\boldsymbol{\times}$***E*** |  |  |  |  |  |
| N stress | 0.52 (0.55) | 6.88 (3.74) | 0.84 (0.64) | 8.81 (4.06) | 51.42 (6.04) |
| N stress +  *Azospirillum* | 9.88 (8.79) | 10.74 (7.33) | 3.32 (4.89) | 12.39 (5.68) | 41.64 (5.49) |

$\sigma_{G}^{2}$: General Combining Ability (GCA), $\sigma_{H}^{2}$: Specific Combining Ability (SCA), $\sigma_{GE}^{2}$: GCA x environment interaction, $\sigma_{HE}^{2}$: SCA x environment interaction, and $\sigma_{\epsilon}^{2}$: residual. Prediction models: GB: GBLUP , GB + G$\times$E: GBLUP + G$\times$E, GK: Gaussian Kernel, and GK + G$\times$E: Gaussian Kernel + G$\times$E.
