# Supplementary material for: Maize responsiveness to Azospirillum brasilense: Insights into genetic control, heterosis and genomic prediction
Source: PLoS One. 2019 Jun 7;14(6):e0217571. doi: 10.1371/journal.pone.0217571 (PMC6555527; doi:10.1371/journal.pone.0217571)
Supplement: S7 Table — σG2: General Combining Ability (GCA); σH2: Specific Combining Ability (SCA); σGE2: GCA x environment interaction; σHE2: SCA x environment interaction; σϵ2: residual by fitting GBLUP (GB), GBLUP+G×E (GB+G×E)), Gaussian Kernel (GK) and Gaussian Kernel + G×E (GK+G×E) models. (DOCX) [file pone.0217571.s010.docx]

**S7 Table. Estimates of variance components and standard deviation (in parentheses) from prediction models for root volume.**

| **Treatment** | $\sigma_{G}^{2}$ | $\sigma_{H}^{2}$ | $\sigma_{GE}^{2}$ | $\sigma_{HE}^{2}$ | $\sigma_{\epsilon}^{2}$ |
| --- | --- | --- | --- | --- | --- |
| ***GB*** |  |  |  |  |  |
| N stress | 1.49 (0.56) | 1.55 (0.45) | - | - | 6.04 (0.65) |
| N stress + *Azospirillum* | 3.49 (1.23) | 1.74 (0.47) | - | - | 5.68 (0.61) |
| ***GB + G***$\boldsymbol{\times}$***E*** |  |  |  |  |  |
| N stress | 1.05 (0.45) | 1.20 (0.41) | 0.68 (0.28) | 1.40 (0.41) | 4.93 (0.72) |
| N stress + *Azospirillum* | 2.69 (1.14) | 1.37 (0.43) | 0.92 (0.40) | 1.36 (0.43) | 4.67 (0.63) |
| ***GK*** |  |  |  |  |  |
| N stress | 0.46 (0.47) | 2.24 (0.92) | - | - | 6.12 (0.66) |
| N stress + *Azospirillum* | 3.39 (1.44) | 1.64 (0.77) | - | - | 6.06 (0.64) |
| ***GK + G***$\boldsymbol{\times}$***E*** |  |  |  |  |  |
| N stress | 0.20 (0.29) | 1.75 (0.83) | 0.19 (0.17) | 1.51 (0.70) | 5.52 (0.71) |
| N stress + *Azospirillum* | 2.95 (1.47) | 1.35 (0.91) | 0.24 (0.22) | 1.39 (0.52) | 5.45 (0.67) |

$\sigma_{G}^{2}$: General Combining Ability (GCA), $\sigma_{H}^{2}$: Specific Combining Ability (SCA), $\sigma_{GE}^{2}$: GCA x environment interaction, $\sigma_{HE}^{2}$: SCA x environment interaction, and $\sigma_{\epsilon}^{2}$: residual. Prediction models: GB: GBLUP , GB + G$\times$E: GBLUP + G$\times$E, GK: Gaussian Kernel, and GK + G$\times$E: Gaussian Kernel + G$\times$E.
