# Supplementary material for: Maize responsiveness to Azospirillum brasilense: Insights into genetic control, heterosis and genomic prediction
Source: PLoS One. 2019 Jun 7;14(6):e0217571. doi: 10.1371/journal.pone.0217571 (PMC6555527; doi:10.1371/journal.pone.0217571)
Supplement: S8 Table — σG2: General Combining Ability (GCA); σH2: Specific Combining Ability (SCA); σGE2: GCA x environment interaction; σHE2: SCA x environment interaction; σϵ2: residual by fitting GBLUP (GB), GBLUP+G×E (GB+G×E), Gaussian Kernel (GK) and Gaussian Kernel + G×E (GK+G×E) models. The values must be multiplied by 10−4 to return to its correct magnitude. (DOCX) [file pone.0217571.s011.docx]

**S8 Table. Estimates of variance components and standard deviation (in parentheses) from prediction models for root average diameter**. The values must be multiplied by 10^-4^ to return to its correct magnitude.

| **Treatment** | $\sigma_{G}^{2}$ | $\sigma_{H}^{2}$ | $\sigma_{GE}^{2}$ | $\sigma_{HE}^{2}$ | $\sigma_{\epsilon}^{2}$ |
| --- | --- | --- | --- | --- | --- |
| ***GB*** |  |  |  |  |  |
| N stress | 2.75 (1.01) | 2.90 (0.78) | - | - | 8.38 (0.93) |
| N stress + *Azospirillum* | 5.69 (2.00) | 2.94 (0.84) | - | - | 11.30 (1.23) |
| ***GB + G***$\boldsymbol{\times}$***E*** |  |  |  |  |  |
| N stress | 2.00 (0.87) | 2.29 (0.72) | 1.12 (0.48) | 2.21 (0.72) | 2.20 (0.96) |
| N stress + *Azospirillum* | 4.55 (1.90) | 2.26 (0.76) | 1.41 (0.63) | 2.30 (0.81) | 9.77 (1.30) |
| ***GK*** |  |  |  |  |  |
| N stress | 0.72 (0.76) | 4.16 (0.15) | - | - | 8.75 (0.99) |
| N stress + *Azospirillum* | 6.21 (2.66) | 2.60 (1.14) | - | - | 11.90 (1.25) |
| ***GK + G***$\boldsymbol{\times}$***E*** |  |  |  |  |  |
| N stress | 0.34 (0.47) | 3.39 (1.50) | 0.36 (0.31) | 2.89 (1.25) | 7.36 (1.07) |
| N stress + *Azospirillum* | 5.83 (2.69) | 2.00 (1.24) | 0.30 (0.30) | 2.62 (1.06) | 10.70 (1.27) |

$\sigma_{G}^{2}$: General Combining Ability (GCA), $\sigma_{H}^{2}$: Specific Combining Ability (SCA), $\sigma_{GE}^{2}$: GCA x environment interaction, $\sigma_{HE}^{2}$: SCA x environment interaction, and $\sigma_{\epsilon}^{2}$: residual. Prediction models: GB: GBLUP , GB + G$\times$E: GBLUP + G$\times$E, GK: Gaussian Kernel, and GK + G$\times$E: Gaussian Kernel + G$\times$E.
