# Supplementary material for: Maize responsiveness to Azospirillum brasilense: Insights into genetic control, heterosis and genomic prediction
Source: PLoS One. 2019 Jun 7;14(6):e0217571. doi: 10.1371/journal.pone.0217571 (PMC6555527; doi:10.1371/journal.pone.0217571)
Supplement: S9 Table — σG2: General Combining Ability (GCA); σH2: Specific Combining Ability (SCA); σGE2: GCA x environment interaction; σHE2: SCA x environment interaction; σϵ2: residual by fitting GBLUP (GB), GBLUP+G×E (GB+G×E), Gaussian Kernel (GK) and Gaussian Kernel + G×E (GK+G×E) models. (DOCX) [file pone.0217571.s012.docx]

**S9 Table. Estimates of variance components and standard deviation (in parentheses) from prediction models for specific root length.**

| **Treatment** | $\sigma_{G}^{2}$ | $\sigma_{H}^{2}$ | $\sigma_{GE}^{2}$ | $\sigma_{HE}^{2}$ | $\sigma_{\epsilon}^{2}$ |
| --- | --- | --- | --- | --- | --- |
| ***GB*** |  |  |  |  |  |
| N stress | 61,037.77  (23,836.21) | 91,452.32  (29,164.38) | - | - | 324,387.30 (36,455.43) |
| N stress + *Azospirillum* | 120,392.80  (44,514.44) | 65,691.42  ( 20,210.64) | - | - | 338,562.50  (35,389.73) |
| ***GB + G***$\boldsymbol{\times}$***E*** |  |  |  |  |  |
| N stress | 42,513.56  (20,385.99) | 75,563.97  ( 28,490.71) | 37,032.54  (15,235.62) | 64,311.60  (27,011.56) | 259,269.70  (38,145.20) |
| N stress + *Azospirillum* | 94,805.63  (41,581.98) | 49,074.92  (17,461.34) | 32,478.37  (15,162.41) | 51,116.82 (18,632.54) | 307,349.20  (37,073.20) |
| ***GK*** |  |  |  |  |  |
| N stress | 9,464.56  (11,067.73) | 120,500.50  (48,872.90) | - | - | 346,739.90 (39794.01) |
| N stress + *Azospirillum* | 116,164.40  (57,104.10) | 69,702.99  (37,091.26) | - | - | 339,307.00  (34,874.36) |
| ***GK + G***$\boldsymbol{\times}$***E*** |  |  |  |  |  |
| N stress | 4,960.72  (6,914.57) | 90,530.97  (47,986.61) | 9,794.91  (10,272.81) | 82,924.39 (42,624.66) | 301,820.00  (43,569.28) |
| N stress + *Azospirillum* | 95,988.61  (59,673.97) | 57,835.98  (43,314.62) | 8,357.87  (12,662.27) | 70,370.65 (31,853.05) | 306,469.00 (36,979.57) |

$\sigma_{G}^{2}$: General Combining Ability (GCA), $\sigma_{H}^{2}$: Specific Combining Ability (SCA), $\sigma_{GE}^{2}$: GCA x environment interaction, $\sigma_{HE}^{2}$: SCA x environment interaction, and $\sigma_{\epsilon}^{2}$: residual. Prediction models: GB: GBLUP , GB + G$\times$E: GBLUP + G$\times$E, GK: Gaussian Kernel, and GK + G$\times$E: Gaussian Kernel + G$\times$E.
