# Supplementary material for: Maize responsiveness to Azospirillum brasilense: Insights into genetic control, heterosis and genomic prediction
Source: PLoS One. 2019 Jun 7;14(6):e0217571. doi: 10.1371/journal.pone.0217571 (PMC6555527; doi:10.1371/journal.pone.0217571)
Supplement: S10 Table — σG2: General Combining Ability (GCA); σH2: Specific Combining Ability (SCA); σGE2: GCA x environment interaction; σHE2: SCA x environment interaction; σϵ2: residual by fitting GBLUP (GB), GBLUP+G×E (GB+G×E), Gaussian Kernel (GK) and Gaussian Kernel + G×E (GK+G×E) models. (DOCX) [file pone.0217571.s013.docx]

| **Treatment** | $\sigma_{G}^{2}$ | $\sigma_{H}^{2}$ | $\sigma_{GE}^{2}$ | $\sigma_{HE}^{2}$ | $\sigma_{\epsilon}^{2}$ |
| --- | --- | --- | --- | --- | --- |
| ***GB*** |  |  |  |  |  |
| N stress | 1,080.95 (431.33) | 1,534.69 (510.57) | - | - | 5,978.15 (678.94) |
| N stress + *Azospirillum* | 1,686.55 (638.28) | 1,181.45 (384.32) | - | - | 5,880.65 (627.51) |
| ***GB + G***$\boldsymbol{\times}$***E*** |  |  |  |  |  |
| N stress | 737.93 (359.18) | 1,298.24 (517.81) | 662.90 (283.02) | 1,145.74 (532.77) | 4,686.34 (736.71) |
| N stress + *Azospirillum* | 1,255.95 (560.06) | 900.37 (340.85) | 563.04 (260.06) | 895.94  (353.42) | 5,235.78 (652.94) |
| ***GK*** |  |  |  |  |  |
| N stress | 218.47 (281.13) | 2,071.81 (939.79) | - | - | 6,414.06  (740.90) |
| N stress + *Azospirillum* | 1,170.52 (834.35) | 1,592.55 (947.50) | - | - | 5,960.32 (636.99) |
| ***GK + G***$\boldsymbol{\times}$***E*** |  |  |  |  |  |
| N stress | 94.04 (137.57) | 1,563.91 (918.66) | 195.77 (229.63) | 1,495.18 (813.64) | 5,561.40 (816.83) |
| N stress + *Azospirillum* | 659.47 (725.73) | 1,511.83 (968.94) | 137.79 (188.96) | 1,578.95 (762.81) | 5,062.23 (699.39) |

**S10 Table. Estimates of variance components and standard deviation (in parentheses) from prediction models for specific root surface area.**

$\sigma_{G}^{2}$: General Combining Ability (GCA), $\sigma_{H}^{2}$: Specific Combining Ability (SCA), $\sigma_{GE}^{2}$: GCA x environment interaction, $\sigma_{HE}^{2}$: SCA x environment interaction, and $\sigma_{\epsilon}^{2}$: residual. Prediction models: GB: GBLUP , GB + G$\times$E: GBLUP + G$\times$E, GK: Gaussian Kernel, and GK + G$\times$E: Gaussian Kernel + G$\times$E.
