# Supplementary material for: Maize responsiveness to Azospirillum brasilense: Insights into genetic control, heterosis and genomic prediction
Source: PLoS One. 2019 Jun 7;14(6):e0217571. doi: 10.1371/journal.pone.0217571 (PMC6555527; doi:10.1371/journal.pone.0217571)
Supplement: S11 Table — (DOCX) [file pone.0217571.s014.docx]

**S11 Table. Adjusted means by maize genotype.**

| **Nº** | **Cross** | **RDM** | **RV** | **RAD** | **SRL** | **SRSA** |  | **RDM** | **RV** | **RAD** | **SRL** | **SRSA** |
| --- | --- | --- | --- | --- | --- | --- | --- | --- | --- | --- | --- | --- |
| 1 | L003xL006 | 1.64 | 22.50 | 0.69 | 4062.94 | 822.06 |  | 1.70 | 21.10 | 0.65 | 3492.76 | 732.07 |
| 2 | L003xL008 | 1.83 | 18.92 | 0.66 | 2862.19 | 600.76 |  | 2.04 | 24.37 | 0.75 | 2357.69 | 615.34 |
| 3 | L003xL014 | 1.41 | 16.96 | 0.62 | 3872.57 | 747.62 |  | 1.58 | 19.43 | 0.66 | 3638.37 | 745.52 |
| 4 | L003xL015 | 1.38 | 16.51 | 0.65 | 3490.28 | 707.18 |  | 1.47 | 17.75 | 0.67 | 3509.88 | 711.52 |
| 5 | L003xL018 | 2.28 | 30.10 | 0.81 | 2330.90 | 637.76 |  | 1.60 | 22.73 | 0.70 | 3746.31 | 844.68 |
| 6 | L003xL023 | 1.40 | 18.02 | 0.61 | 4735.04 | 882.43 |  | 1.23 | 17.86 | 0.66 | 4936.65 | 964.54 |
| 7 | L003xL026 | 1.69 | 19.46 | 0.70 | 2817.10 | 632.88 |  | 1.83 | 22.77 | 0.73 | 2615.36 | 649.51 |
| 8 | L003xL032 | 1.91 | 26.18 | 0.74 | 3069.53 | 735.55 |  | 1.84 | 24.74 | 0.75 | 2853.39 | 716.85 |
| 9 | L003xL034 | 1.58 | 21.07 | 0.71 | 3258.61 | 713.05 |  | 1.35 | 16.91 | 0.66 | 3505.20 | 727.47 |
| 10 | L003xL041 | 1.39 | 21.31 | 0.71 | 2859.95 | 706.73 |  | 1.73 | 22.52 | 0.68 | 3585.07 | 767.99 |
| 11 | L003xL047 | 1.65 | 22.73 | 0.78 | 2874.60 | 738.09 |  | 1.38 | 18.84 | 0.70 | 3572.14 | 795.85 |
| 12 | L003xL049 | 1.45 | 18.22 | 0.65 | 3770.87 | 767.06 |  | 1.39 | 17.27 | 0.63 | 4058.01 | 788.34 |
| 13 | L003xL052 | 1.81 | 22.66 | 0.70 | 2972.48 | 672.65 |  | 1.51 | 19.71 | 0.70 | 3465.37 | 767.39 |
| 14 | L006xL008 | 1.54 | 22.71 | 0.72 | 3790.76 | 872.86 |  | 1.38 | 20.17 | 0.72 | 3650.39 | 824.33 |
| 15 | L006xL011 | 1.68 | 23.09 | 0.69 | 3631.83 | 799.38 |  | 1.33 | 18.23 | 0.66 | 4117.35 | 857.45 |
| 16 | L006xL014 | 1.47 | 20.73 | 0.64 | 4544.32 | 900.17 |  | 1.28 | 18.43 | 0.68 | 4078.75 | 869.70 |
| 17 | L006xL015 | 1.74 | 18.92 | 0.66 | 3271.28 | 660.35 |  | 1.80 | 20.37 | 0.69 | 2817.39 | 631.27 |
| 18 | L006xL023 | 1.79 | 24.60 | 0.68 | 3855.28 | 793.02 |  | 1.62 | 21.86 | 0.69 | 3646.09 | 796.09 |
| 19 | L006xL026 | 1.70 | 22.84 | 0.71 | 3350.06 | 753.17 |  | 1.48 | 19.49 | 0.65 | 4472.40 | 861.25 |
| 20 | L006xL038 | 1.57 | 20.21 | 0.71 | 3049.81 | 703.96 |  | 1.58 | 19.78 | 0.72 | 2728.97 | 657.76 |
| 21 | L006xL047 | 1.85 | 21.83 | 0.62 | 3741.46 | 714.88 |  | 1.31 | 17.58 | 0.66 | 4044.07 | 826.20 |
| 22 | L006xL049 | 1.72 | 21.58 | 0.73 | 2765.74 | 656.71 |  | 1.32 | 17.99 | 0.67 | 4078.88 | 841.82 |
| 23 | L006xL052 | 1.46 | 21.72 | 0.70 | 3994.66 | 877.02 |  | 1.64 | 21.96 | 0.69 | 3455.45 | 770.67 |
| 24 | L008xL011 | 1.92 | 22.91 | 0.63 | 3670.06 | 731.98 |  | 1.47 | 21.04 | 0.68 | 4117.25 | 875.74 |
| 25 | L008xL015 | 1.20 | 15.90 | 0.62 | 3565.75 | 742.73 |  | 1.54 | 19.72 | 0.71 | 2880.38 | 677.41 |
| 26 | L008xL018 | 1.78 | 24.90 | 0.81 | 2572.49 | 679.78 |  | 1.75 | 24.13 | 0.71 | 3505.05 | 803.26 |
| 27 | L008xL023 | 1.26 | 16.93 | 0.63 | 5250.48 | 992.42 |  | 1.14 | 16.01 | 0.66 | 4326.81 | 880.17 |
| 28 | L008xL026 | 1.63 | 21.44 | 0.69 | 3531.20 | 744.15 |  | 1.36 | 16.95 | 0.64 | 4156.70 | 801.64 |
| 29 | L008xL032 | 1.51 | 20.69 | 0.74 | 3742.43 | 813.23 |  | 1.20 | 18.66 | 0.68 | 4778.72 | 1017.09 |
| 30 | L008xL034 | 1.40 | 20.58 | 0.69 | 4326.66 | 908.76 |  | 2.06 | 24.38 | 0.75 | 2432.55 | 609.79 |
| 31 | L008xL041 | 1.60 | 19.82 | 0.67 | 3370.50 | 721.37 |  | 1.03 | 13.92 | 0.63 | 4936.69 | 930.15 |
| 32 | L008xL047 | 1.58 | 19.40 | 0.63 | 4042.70 | 793.17 |  | 1.69 | 23.31 | 0.69 | 3565.32 | 786.59 |
| 33 | L008xL048 | 1.79 | 25.43 | 0.71 | 3859.96 | 843.24 |  | 1.40 | 17.92 | 0.62 | 4186.42 | 804.26 |
| 34 | L008xL049 | 0.97 | 12.78 | 0.62 | 4478.39 | 860.53 |  | 1.22 | 15.22 | 0.65 | 4126.15 | 827.35 |
| 35 | L008xL052 | 1.94 | 24.69 | 0.79 | 2259.90 | 612.60 |  | 1.51 | 21.36 | 0.77 | 2969.62 | 752.04 |
| 36 | L008xL056 | 1.08 | 14.27 | 0.64 | 4391.17 | 874.05 |  | 1.20 | 16.22 | 0.67 | 4106.15 | 827.91 |
| 37 | L011xL014 | 1.48 | 20.18 | 0.66 | 4234.01 | 871.02 |  | 1.34 | 18.07 | 0.65 | 4239.51 | 846.70 |
| 38 | L011xL015 | 1.82 | 21.41 | 0.72 | 2678.36 | 625.01 |  | 1.87 | 23.98 | 0.69 | 3148.10 | 713.78 |
| 39 | L011xL018 | 1.65 | 23.54 | 0.70 | 3900.84 | 837.34 |  | 1.64 | 22.86 | 0.68 | 4092.89 | 854.70 |
| 40 | L011xL023 | 1.48 | 21.93 | 0.65 | 4980.92 | 992.87 |  | 1.45 | 22.27 | 0.70 | 4124.19 | 915.93 |
| 41 | L011xL026 | 2.02 | 25.82 | 0.69 | 3218.91 | 698.50 |  | 1.32 | 18.34 | 0.68 | 4074.17 | 867.99 |
| 42 | L011xL032 | 1.75 | 24.67 | 0.67 | 4593.52 | 913.68 |  | 2.00 | 27.23 | 0.69 | 3986.38 | 868.64 |
| 43 | L011xL034 | 1.70 | 29.48 | 0.73 | 4324.15 | 988.84 |  | 1.53 | 21.86 | 0.68 | 4097.26 | 870.33 |
| 44 | L011xL038 | 1.69 | 23.23 | 0.69 | 3624.36 | 792.94 |  | 1.69 | 22.42 | 0.71 | 3181.83 | 741.72 |
| 45 | L011xL047 | 1.31 | 18.03 | 0.68 | 3842.97 | 818.22 |  | 1.04 | 15.21 | 0.62 | 5385.28 | 1011.11 |
| 46 | L011xL056 | 1.28 | 18.34 | 0.64 | 4594.55 | 872.99 |  | 1.11 | 14.94 | 0.63 | 4811.65 | 922.43 |
| 47 | L014xL015 | 1.41 | 16.21 | 0.63 | 3730.50 | 704.45 |  | 1.71 | 19.44 | 0.68 | 3013.70 | 649.85 |
| 48 | L014xL018 | 1.72 | 21.36 | 0.68 | 3585.88 | 750.53 |  | 1.89 | 24.62 | 0.72 | 3021.39 | 711.20 |
| 49 | L014xL023 | 1.41 | 21.14 | 0.63 | 5421.40 | 1002.34 |  | 1.09 | 16.74 | 0.66 | 5122.87 | 1036.94 |
| 50 | L014xL026 | 1.53 | 18.40 | 0.65 | 3639.08 | 739.33 |  | 1.67 | 20.09 | 0.67 | 3398.31 | 727.07 |
| 51 | L014xL032 | 1.68 | 18.93 | 0.59 | 4108.80 | 751.26 |  | 1.24 | 18.73 | 0.71 | 3884.26 | 877.55 |
| 52 | L014xL034 | 1.23 | 15.88 | 0.59 | 5102.94 | 921.51 |  | 0.99 | 14.31 | 0.63 | 4970.50 | 936.41 |
| 53 | L014xL038 | 1.62 | 18.32 | 0.64 | 3516.61 | 711.95 |  | 1.55 | 22.77 | 0.74 | 3349.94 | 790.45 |
| 54 | L014xL041 | 1.62 | 18.81 | 0.60 | 4386.05 | 798.52 |  | 1.54 | 18.97 | 0.64 | 3760.37 | 749.60 |
| 55 | L014xL047 | 1.35 | 17.82 | 0.60 | 4946.16 | 898.60 |  | 1.30 | 18.69 | 0.69 | 3770.87 | 820.78 |
| 56 | L014xL048 | 1.47 | 17.87 | 0.63 | 3939.70 | 762.47 |  | 1.30 | 15.43 | 0.57 | 3854.11 | 900.19 |
| 57 | L014xL049 | 0.95 | 12.75 | 0.63 | 4652.45 | 895.62 |  | 0.49 | 8.70 | 0.57 | 5333.34 | 1005.78 |
| 58 | L014xL056 | 1.36 | 16.19 | 0.67 | 3338.08 | 709.34 |  | 1.40 | 19.94 | 0.69 | 3914.88 | 842.68 |
| 59 | L015xL018 | 2.08 | 23.65 | 0.67 | 2829.66 | 609.63 |  | 1.65 | 19.96 | 0.69 | 3017.32 | 661.88 |
| 60 | L015xL023 | 1.40 | 18.18 | 0.64 | 4222.94 | 829.86 |  | 1.60 | 20.96 | 0.66 | 4144.38 | 823.27 |
| 61 | L015xL032 | 1.99 | 24.50 | 0.71 | 2831.14 | 647.57 |  | 1.71 | 20.15 | 0.71 | 2808.22 | 648.57 |
| 62 | L015xL034 | 1.41 | 18.30 | 0.67 | 3691.78 | 773.04 |  | 1.36 | 16.86 | 0.64 | 4167.24 | 810.28 |
| 63 | L015xL038 | 1.93 | 23.52 | 0.69 | 3111.59 | 694.58 |  | 1.52 | 19.01 | 0.66 | 3618.62 | 756.73 |
| 64 | L015xL041 | 1.44 | 16.72 | 0.61 | 4398.82 | 825.82 |  | 1.26 | 15.52 | 0.65 | 3725.68 | 748.66 |
| 65 | L015xL047 | 1.08 | 14.73 | 0.60 | 5499.13 | 996.04 |  | 1.33 | 17.46 | 0.65 | 4050.87 | 813.33 |
| 66 | L015xL052 | 1.80 | 22.45 | 0.70 | 2928.34 | 663.12 |  | 1.54 | 18.75 | 0.70 | 2976.62 | 672.59 |
| 67 | L015xL055 | 1.29 | 17.09 | 0.63 | 4356.81 | 846.59 |  | 1.11 | 15.65 | 0.60 | 5748.94 | 1041.16 |
| 68 | L015xL056 | 1.07 | 12.46 | 0.58 | 4105.57 | 760.71 |  | 1.45 | 16.84 | 0.67 | 3189.21 | 682.27 |
| 69 | L018xL023 | 1.44 | 20.31 | 0.62 | 4963.11 | 942.15 |  | 1.35 | 20.06 | 0.64 | 4913.91 | 967.31 |
| 70 | L018xL032 | 2.32 | 30.75 | 0.82 | 2188.16 | 618.80 |  | 1.65 | 26.34 | 0.76 | 2957.24 | 728.86 |
| 71 | L018xL038 | 1.70 | 24.95 | 0.75 | 3366.41 | 816.68 |  | 2.08 | 27.41 | 0.78 | 2473.00 | 662.50 |
| 72 | L018xL041 | 2.02 | 24.19 | 0.69 | 2984.36 | 669.09 |  | 1.61 | 22.88 | 0.69 | 3923.70 | 859.68 |
| 73 | L018xL055 | 2.02 | 27.73 | 0.68 | 3883.28 | 817.47 |  | 1.62 | 22.36 | 0.67 | 4343.13 | 901.40 |
| 74 | L018xL056 | 1.42 | 17.81 | 0.59 | 4907.62 | 854.94 |  | 1.30 | 18.17 | 0.64 | 4981.05 | 938.31 |
| 75 | L023xL026 | 1.25 | 17.02 | 0.68 | 4058.95 | 840.36 |  | 1.90 | 24.53 | 0.70 | 3717.02 | 795.05 |
| 76 | L023xL032 | 1.61 | 22.62 | 0.69 | 3836.93 | 839.73 |  | 1.74 | 26.39 | 0.73 | 3667.38 | 840.70 |
| 77 | L023xL034 | 1.71 | 23.94 | 0.73 | 3965.04 | 852.47 |  | 1.27 | 21.22 | 0.70 | 4922.15 | 1014.41 |
| 78 | L023xL038 | 1.89 | 26.19 | 0.77 | 2733.11 | 700.23 |  | 1.67 | 24.11 | 0.70 | 4115.90 | 874.08 |
| 79 | L023xL041 | 1.51 | 21.70 | 0.70 | 3805.71 | 844.01 |  | 1.28 | 19.24 | 0.62 | 5222.11 | 987.87 |
| 80 | L023xL047 | 1.52 | 19.93 | 0.64 | 4142.02 | 817.25 |  | 1.39 | 19.16 | 0.68 | 4116.60 | 878.28 |
| 81 | L023xL048 | 1.58 | 20.35 | 0.67 | 3603.94 | 757.87 |  | 1.22 | 17.06 | 0.64 | 4614.70 | 915.91 |
| 82 | L023xL049 | 1.27 | 15.21 | 0.59 | 5117.84 | 902.95 |  | 1.36 | 17.87 | 0.67 | 4310.44 | 848.47 |
| 83 | L023xL055 | 1.41 | 19.39 | 0.62 | 4864.35 | 927.02 |  | 1.32 | 20.60 | 0.65 | 5519.08 | 1098.49 |
| 84 | L023xL056 | 1.28 | 18.57 | 0.64 | 3894.33 | 977.22 |  | 1.43 | 18.02 | 0.60 | 4825.71 | 877.43 |
| 85 | L026xL032 | 2.02 | 26.12 | 0.75 | 2590.22 | 656.35 |  | 1.34 | 18.54 | 0.68 | 4247.41 | 873.97 |
| 86 | L026xL038 | 1.64 | 22.94 | 0.73 | 3254.49 | 760.00 |  | 1.33 | 18.76 | 0.69 | 3732.13 | 814.83 |
| 87 | L026xL047 | 1.14 | 15.53 | 0.63 | 4793.13 | 926.97 |  | 1.47 | 22.86 | 0.71 | 4246.83 | 914.37 |
| 88 | L032xL034 | 1.58 | 20.76 | 0.63 | 4389.94 | 850.51 |  | 2.03 | 25.71 | 0.72 | 3644.59 | 818.10 |
| 89 | L032xL038 | 1.88 | 25.86 | 0.77 | 2922.97 | 714.77 |  | 1.74 | 22.13 | 0.72 | 3083.76 | 727.50 |
| 90 | L032xL047 | 1.45 | 19.77 | 0.63 | 4745.90 | 935.32 |  | 1.65 | 22.62 | 0.67 | 3982.29 | 846.17 |
| 91 | L032xL052 | 1.76 | 22.63 | 0.68 | 3402.14 | 740.09 |  | 1.45 | 18.94 | 0.67 | 3854.36 | 817.83 |
| 92 | L034xL041 | 2.04 | 21.79 | 0.65 | 2896.68 | 602.15 |  | 1.44 | 18.15 | 0.65 | 3765.75 | 767.45 |
| 93 | L034xL047 | 1.53 | 19.57 | 0.65 | 4096.05 | 801.44 |  | 1.49 | 20.00 | 0.71 | 3747.17 | 810.05 |
| 94 | L034xL049 | 1.36 | 17.55 | 0.64 | 4084.21 | 803.17 |  | 1.59 | 17.94 | 0.65 | 3290.47 | 673.42 |
| 95 | L034xL052 | 1.59 | 20.81 | 0.75 | 2909.21 | 702.21 |  | 1.56 | 22.49 | 0.72 | 3581.56 | 809.14 |
| 96 | L034xL055 | 1.51 | 18.05 | 0.61 | 4064.57 | 767.58 |  | 1.57 | 20.12 | 0.64 | 4082.25 | 811.11 |
| 97 | L034xL056 | 1.57 | 18.70 | 0.61 | 4205.08 | 768.51 |  | 1.35 | 18.59 | 0.66 | 3956.47 | 823.27 |
| 98 | L038xL047 | 1.04 | 15.84 | 0.64 | 5384.42 | 1052.70 |  | 1.07 | 16.01 | 0.68 | 4308.39 | 902.51 |
| 99 | L038xL049 | 1.54 | 21.30 | 0.75 | 2976.00 | 727.73 |  | 1.26 | 15.11 | 0.65 | 3641.81 | 741.72 |
| 100 | L038xL052 | 1.89 | 23.20 | 0.75 | 2600.99 | 640.26 |  | 1.34 | 18.94 | 0.69 | 4141.50 | 862.17 |
| 101 | L038xL055 | 1.24 | 17.32 | 0.63 | 3810.84 | 796.08 |  | 1.56 | 21.96 | 0.71 | 3514.92 | 805.17 |
| 102 | L038xL056 | 1.74 | 22.02 | 0.71 | 3057.36 | 697.01 |  | 1.70 | 20.02 | 0.69 | 2929.53 | 657.51 |
| 103 | L041xL047 | 1.33 | 17.01 | 0.59 | 4949.78 | 869.65 |  | 1.29 | 16.98 | 0.59 | 5367.81 | 937.99 |
| 104 | L041xL049 | 1.45 | 18.14 | 0.66 | 3588.58 | 747.87 |  | 1.18 | 15.55 | 0.66 | 4190.95 | 842.16 |
| 105 | L041xL056 | 1.35 | 16.76 | 0.62 | 4144.09 | 797.90 |  | 1.29 | 15.43 | 0.62 | 3931.40 | 751.09 |
| 106 | L047xL048 | 1.31 | 16.28 | 0.60 | 4438.91 | 823.52 |  | 1.46 | 19.26 | 0.63 | 4359.83 | 846.56 |
| 107 | L047xL052 | 1.49 | 19.94 | 0.71 | 3432.26 | 778.12 |  | 1.36 | 18.34 | 0.68 | 3823.83 | 803.70 |
| 108 | L047xL055 | 1.71 | 22.37 | 0.58 | 5230.21 | 913.79 |  | 1.33 | 17.42 | 0.55 | 6352.01 | 1027.81 |
| 109 | L047xL056 | 1.11 | 14.84 | 0.60 | 5015.37 | 925.19 |  | 1.32 | 15.99 | 0.63 | 4130.40 | 788.46 |
| 110 | L048xL049 | 1.04 | 12.76 | 0.61 | 4187.08 | 797.13 |  | 0.98 | 12.36 | 0.62 | 4253.34 | 804.32 |
| 111 | L048xL052 | 1.78 | 21.17 | 0.72 | 2628.82 | 619.42 |  | 1.88 | 20.58 | 0.68 | 2585.44 | 585.85 |
| 112 | L048xL055 | 1.06 | 14.67 | 0.61 | 5055.00 | 936.83 |  | 1.30 | 15.91 | 0.62 | 4158.65 | 794.56 |
| 113 | L048xL056 | 1.06 | 13.72 | 0.58 | 5642.37 | 968.41 |  | 1.27 | 17.30 | 0.63 | 4724.06 | 887.40 |
| 114 | L049xL052 | 1.81 | 23.10 | 0.73 | 2877.04 | 676.63 |  | 1.85 | 22.54 | 0.73 | 2551.40 | 622.56 |
| 115 | L049xL056 | 1.52 | 17.41 | 0.66 | 3065.87 | 642.33 |  | 1.28 | 16.14 | 0.64 | 3778.89 | 766.01 |
| 116 | L052xL055 | 1.49 | 19.74 | 0.63 | 4475.85 | 863.07 |  | 1.54 | 19.31 | 0.65 | 3752.22 | 764.80 |
| 117 | L052xL056 | 1.48 | 19.79 | 0.69 | 3427.08 | 757.50 |  | 1.42 | 18.35 | 0.66 | 3712.35 | 773.49 |
| 118 | L055xL056 | 1.55 | 19.81 | 0.62 | 4339.08 | 829.83 |  | 1.46 | 18.80 | 0.61 | 4609.93 | 855.72 |

RDM: root dry mass, RV: root volume, RAD: root average diameter, SRL: specific root length, and SRSA: specific root surface area.
